# Classification of High-Grade Serous Ovarian Cancer Using Tumor Morphologic Characteristics

**DOI:** 10.1001/jamanetworkopen.2022.36626

**Published:** 2022-10-14

**Authors:** Katelyn F. Handley, Travis T. Sims, Nicholas W. Bateman, Deanna Glassman, Katherine I. Foster, Sanghoon Lee, Jun Yao, Hui Yao, Bryan M. Fellman, Jinsong Liu, Zhen Lu, Kelly A. Conrads, Brian L. Hood, Waleed Barakat, Li Zhao, Jianhua Zhang, Shannon N. Westin, Joseph Celestino, Kelly M. Rangel, Sunil Badal, Igor Pereira, Prahlad T. Ram, George L. Maxwell, Livia S. Eberlin, P. Andrew Futreal, Robert C. Bast, Nicole D. Fleming, Thomas P. Conrads, Anil K. Sood

**Affiliations:** 1 Department of Gynecologic Oncology and Reproductive Medicine, The University of Texas MD Anderson Cancer Center, Houston; 2 Division of Gynecologic Oncology, Department of Obstetrics and Gynecology, Morsani College of Medicine, University of South Florida, Tampa; 3 Department of Gynecologic Oncology, H. Lee Moffitt Cancer Center and Research Institute, Tampa, Florida; 4 Women’s Health Integrated Research Center at Inova Health System, Women's Service Line, Inova Health System, Falls Church, Virginia; 5 Gynecologic Cancer Center of Excellence, Henry M. Jackson Foundation, Department of Gynecologic Surgery and Obstetrics, Uniformed Services University and Walter Reed National Military Medical Center, Bethesda, Maryland; 6 Department of Systems Biology, The University of Texas MD Anderson Cancer Center, Houston; 7 Department of Molecular and Cellular Oncology, The University of Texas MD Anderson Cancer Center, Houston; 8 Department of Bioinformatics and Computational Biology, The University of Texas MD Anderson Cancer Center, Houston; 9 Department of Biostatistics, The University of Texas MD Anderson Cancer Center, Houston; 10 Department of Pathology, The University of Texas MD Anderson Cancer Center, Houston; 11 Department of Experimental Therapeutics, The University of Texas MD Anderson Cancer Center, Houston; 12 Department of Genomic Medicine, The University of Texas MD Anderson Cancer Center, Houston; 13 Department of Chemistry, The University of Texas at Austin, Austin; 14 Department of Surgery, Baylor College of Medicine, Houston, Texas

## Abstract

**Importance:**

Despite similar histologic appearance among high-grade serous ovarian cancers (HGSOCs), clinical observations suggest vast differences in gross appearance. There is currently no systematic framework by which to classify HGSOCs according to their gross morphologic characteristics.

**Objective:**

To develop and characterize a gross morphologic classification system for HGSOC.

**Design, Setting, and Participants:**

This cohort study included patients with suspected advanced-stage ovarian cancer who presented between April 1, 2013, and August 5, 2016, to the University of Texas MD Anderson Cancer Center, a large referral center. Patients underwent laparoscopic assessment of disease burden before treatment and received a histopathologic diagnosis of HGSOC. Researchers assigning morphologic subtype and performing molecular analyses were blinded to clinical outcomes. Data analysis was performed between April 2020 and November 2021.

**Exposures:**

Gross tumor morphologic characteristics.

**Main Outcomes and Measures:**

Clinical outcomes and multiomic profiles of representative tumor samples of type I or type II morphologic subtypes were compared.

**Results:**

Of 112 women (mean [SD] age 62.7 [9.7] years) included in the study, most patients (84% [94]) exhibited a predominant morphologic subtype and many (63% [71]) had a uniform morphologic subtype at all involved sites. Compared with those with uniform type I morphologic subtype, patients with uniform type II morphologic subtype were more likely to have a favorable Fagotti score (83% [19 of 23] vs 46% [22 of 48]; *P* = .004) and thus to be triaged to primary tumor reductive surgery. Similarly, patients with uniform type II morphologic subtype also had significantly higher mean (SD) estimated blood loss (639 [559; 95% CI, 391-887] mL vs 415 [527; 95% CI, 253-577] mL; *P* = .006) and longer mean (SD) operative time (408 [130; 95% CI, 350-466] minutes vs 333 [113; 95% CI, 298-367] minutes; *P* = .03) during tumor reductive surgery. Type I tumors had enrichment of epithelial-mesenchymal transition (false discovery rate [FDR] q-value, 3.10 × 10^−24^), hypoxia (FDR q-value, 1.52 × 10^−5^), and angiogenesis pathways (FDR q-value, 2.11 × 10^−2^), whereas type II tumors had enrichment of pathways related to MYC signaling (FDR q-value, 2.04 × 10^−9^) and cell cycle progression (FDR q-value, 1.10 × 10^−5^) by integrated proteomic and transcriptomic analysis. Abundances of metabolites and lipids also differed between the 2 morphologic subtypes.

**Conclusions and Relevance:**

This study identified 2 novel, gross morphologic subtypes of HGSOC, each with unique clinical features and molecular signatures. The findings may have implications for triaging patients to surgery or chemotherapy, identifying outcomes, and developing tailored therapeutic strategies.

## Key Points

**Question:**

Do observed variations in gross morphologic characteristics among high-grade serous ovarian cancers (HGSOCs) represent clinically relevant subtypes?

**Findings:**

In this cohort study of 112 women with advanced-stage ovarian cancer, HGSOCs were reliably classified into 2 morphologic subtypes. Type I and type II morphologic subtypes differed with respect to clinical outcomes as well as transcriptomic, proteomic, and metabolomic profiles.

**Meaning:**

Findings of this study suggest that the molecular and metabolic signatures associated with gross morphologic characteristics of HGSOCs have implications for therapeutic strategies and outcomes.

## Teaser

This cohort study assesses the clinical outcomes and multiomic profiles of tumor specimens from women with advanced-stage ovarian cancer.

## Introduction

Despite the similar histologic appearances of high-grade serous ovarian cancers (HGSOCs), clinical observations point to marked differences in their gross appearances. Some patients have numerous small nodules, whereas others have bulkier disease; some patients have widespread disease, whereas others have more localized disease. However, a systematic framework for classifying such gross morphologic differences does not exist. The implementation of a laparoscopic triage algorithm for patients with advanced HGSOC enabled us to prospectively obtain detailed video images of HGSOC that could be analyzed to identify morphologic subtypes.

In this study, we aimed to develop and characterize a gross morphologic classification system for HGSOC. Specifically, we assessed whether HGSOC can be reliably divided into distinct gross morphologic subtypes and whether such subtypes have different clinical outcomes and molecular features.

## Methods

This cohort study was approved by The University of Texas MD Anderson Cancer Center Institutional Review Board and Quality Improvement Board. All patients provided written informed consent before sample collection. We followed the Strengthening the Reporting of Observational Studies in Epidemiology (STROBE) reporting guideline.^1^ An overview of the study is provided in eFigure 1 in the Supplement.

### Study Design and Patient Selection

Between April 1, 2013, and August 5, 2016, patients with suspected advanced HGSOC who were deemed to be possible candidates for upfront cytoreduction underwent a laparoscopic assessment of disease burden before treatment, as described previously,^2^ at the University of Texas MD Anderson Cancer Center, a large referral center in Houston, Texas. After laparoscopic assessment, patients with a Fagotti score (range, 0-14),^3,4,5^ or predictive index value (PIV), lower than 8 (indicating likelihood of optimal cytoreduction) were offered primary tumor reductive surgery, and patients with a PIV of 8 or higher (indicating low likelihood of optimal cytoreduction) were recommended neoadjuvant chemotherapy followed by interval tumor reductive surgery. Demographic and clinical data, laparoscopic videos, and pretreatment tissue samples from primary and metastatic sites were collected prospectively. The eMethods in the Supplement provides additional details. Self-reported race and ethnicity data are included in eTable 1 in the Supplement.

### Morphologic Review

**Figure 1.**
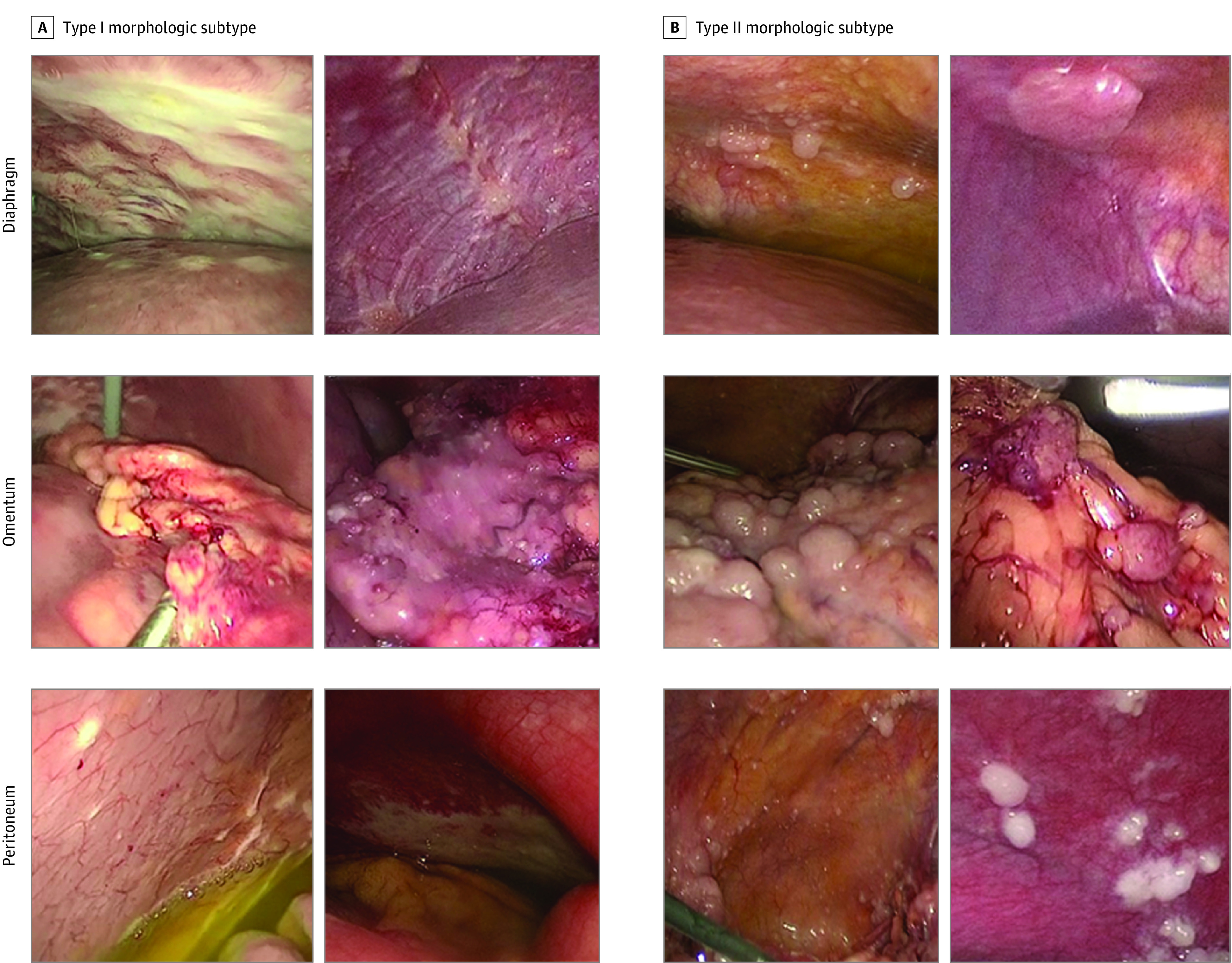
Representative Laparoscopic Images Examples of type I and type II morphologic subtype of the diaphragm, omentum, and peritoneum.

Video recordings of laparoscopic assessments of disease burden in patients with advanced-stage HGSOC were retrospectively reviewed by 3 of us (K.F.H., T.T.S., and D.G.) who were blinded to clinical outcomes. Consensus definitions of 2 distinct types of HGSOC based on gross morphologic characteristics were developed (Table 1 and Figure 1). Four sites (diaphragm, omentum, peritoneum, and pelvis) were assessed, and any disease at these sites was classified as type I (defined as deep, infiltrative disease with distortion of surrounding tissue) or type II (defined as superficial, exophytic disease bordered by normal tissue). Morphologic subtype was considered to be uniform if all involved metastatic sites were classified as the same subtype (ie, of the 4 evaluated sites, wherever metastases were present, all sites were classified as either morphologic type I or type II). Morphologic subtype was considered to be predominant if most of the involved metastatic sites were classified as the same subtype. This definition included patients with uniform morphologic subtypes and those with 1 outlier site identified as the other subtype.

**Table 1.** Characteristics of Type I and Type II Morphologic Subtypes

Subtype	Description
Type I	Deep, infiltrative appearance, including omental caking
	Large, raised plaques
	Miliary lesions
	Macular or papular
	Distortion or retraction of surrounding tissue
	Stellate
	Agglutinated
Type II	Superficial appearance
	Exophytic nodules or patches; pedunculated
	Irregular tissue
	Frond-like undulating surfaces
	Bordered by normal tissue
	Distinct edges
	Hypervascular

### Multiomic Analysis of Patient-Derived Tumor Samples

Frozen primary and metastatic tumor tissues were analyzed by quantitative mass spectrometry (MS). Peptide and global protein-level identifications were generated by searching raw data files with a publicly available, nonredundant human proteome database. Reverse phase protein arrays were also performed on frozen tissues. Data were examined with NetWalker software (Zhang Lab at Baylor College of Medicine). The median relative protein expression was compared between patients with uniform type I and uniform type II morphologic subtypes. For RNA sequencing, total RNA from frozen samples was obtained and sequenced.

Principal component analysis and unsupervised clustering were performed using the top 3000 most variably expressed genes. Gene set enrichment analysis was performed. Functional categories of differentially expressed genes were obtained using the Database for Annotation, Visualization and Integrated Discovery (Laboratory of Human Retrovirology and Immunoinformatics).^6,7^ Differential analyses of merged, global proteome, or transcriptome matrices were performed using the LIMMA (linear models for microarray data) package, version 3.8,^8^ in R, version 3.5.2 (R Foundation for Statistical Computing). Pathway analysis was performed using hallmark gene sets in the Molecular Signatures Database, version 7.4 (UC San Diego and Broad Institute). Immune-profiling analysis was performed as described previously.^9^ Abundances and distributions of immune infiltrates in the predominant type I tissues were compared with those in the predominant type II tissues. DESI (desorption electrospray ionization)–MS imaging was conducted on primary and metastatic tissue sections. BioMap (Novartis) and MSiReader (North Carolina State University) software were used to plot the images. High mass accuracy and tandem MS measurements were used to identify the ions. DESI-MS data from the tumor regions identified by pathology review were extracted from the ion images using the MSiReader software.^10^ Ion peaks that appeared in more than 10% of the pixels were used for statistical analysis. Significant ion features were identified by significance analysis of microarrays (SAM), and tentative identification was provided for those with a false discovery rate (FDR) of less than 5%.^11^ Additional details on these assays are provided in the eMethods in the Supplement.

### Statistical Analysis

Summary statistics, such as means, SDs, ranges, frequencies, and percentages, were used to describe the study population. Clinical and demographic variables were assessed and compared by morphologic subtype using χ^2^ test, Fisher exact test, analysis of variance, or Kruskal-Wallis as appropriate. Agreement between physician assessment of morphologic classification was quantified with a κ statistic.

Except where specified otherwise, a 2-sided *P* < .05 was considered to be statistically significant. The eMethods in the Supplement provides additional details. Data analysis was performed between April 2020 and November 2021.

## Results

### Classification and Definition of Morphologic Subtypes

This study included 112 patients with HGSOC who underwent laparoscopic assessment of disease burden before treatment. Patients were women with a mean (SD) age of 62.7 (9.7) years who had ovarian (87% [97 of 112]), fallopian tube (5% [6]), or primary peritoneal (8% [9]) carcinoma and were predominantly diagnosed with stage IIIC disease (84% [94]). Detailed baseline characteristics are presented in eTable 1 in the Supplement. Seventy-one patients (63%) exhibited uniform morphologic subtype at all involved metastatic sites. Ninety-four patients (84%) exhibited a predominant morphologic subtype, including those with uniform morphologic subtype. A review of 341 images from 39 patients by a third physician yielded an interrater concordance of 84% with a κ statistic of 0.64, which is considered to be substantial agreement.^12^

Clinical outcomes of the patients by morphologic subtype are presented in Table 2. Compared with those with uniform type I morphologic subtype, patients with uniform type II were more likely to have a laparoscopy-based PIV lower than 8 (83% [19 of 23; 95% CI, 61%-95%] vs 46% [22 of 48; 95% CI, 31%-61%]; *P* = .004) and thus were more likely to be triaged to primary tumor reductive surgery. Among patients who received neoadjuvant chemotherapy, patients with uniform type I had a higher rate of excellent response than those with uniform type II, but this difference was not significant. Compared with patients with uniform type I, patients with uniform type II had a longer mean (SD) operative time during tumor reductive surgery (408 [130; 95% CI, 350-466] minutes vs 333 [113; 95% CI, 298-367] minutes; *P* = .03) and significantly higher mean (SD) estimated blood loss (639 [559; 95% CI, 391-887] mL vs 415 [527; 95% CI, 253-577] mL; *P* = .006). The findings also held true for patients with predominant type II morphologic subtype (mean [SD] operative time: 400 [133] minutes vs 330 [110] minutes, *P* = .02; mean [SD] estimated blood loss: 717 [673] mL vs 406 [492] mL, *P* = .001). However, there was no significant difference in the amount of residual disease. Patients with predominant type II disease had a significantly longer mean (SD) hospital stay than those with type I (7.8 [7.6] days vs 6.1 [6.7] days; *P* = .03).

**Table 2.** Select Clinical Outcomes According to Predominant or Uniform Type I and Type II Morphologic Subtype

Characteristic	Predominant morphologic subtype			Uniform morphologic subtype		
	Type I (n = 57)	Type II (n = 37)	*P* value	Type I (n = 48)	Type II (n = 23)	*P* value
Response to NACT, No. (%)						
Excellent	NA	NA	NA	17 (63)	3 (38)	.25
Poor	NA	NA		2 (7)	2 (25)	
Nonclassifiable	NA	NA		8 (30)	3 (38)	
PIV, No. (%)						
<8	27 (47)	25 (68)	.05	22 (46)	19 (83)	.004
≥8	30 (53)	12 (32)		26 (54)	4 (17)	
Postlaparoscopy treatment, No. (%)						
NACT	32 (56)	18 (49)	.48	27 (56)	8 (35)	.09
pTRS	25 (44)	19 (51)		21 (44)	15 (65)	
TRS, No. (%)						
Not performed	4 (7)	2 (5)	.84	4 (8)	1 (4)	.28
Primary	25 (44)	19 (51)		21 (44)	15 (65)	
Interval	28 (49)	16 (43)		23 (48)	7 (30)	
Operative time, min						
No.	52	35	.02	43	20	.03
Mean (SD)	330 (110)	400 (133)		333 (113)	408 (130)	
Median (range)	326 (140-711)	393 (222-717)		322 (140-711)	396 (222-717)	
Estimated blood loss, mL						
No.	52	35	.001	43	22	.006
Mean (SD)	406 (492)	717 (673)		415 (527)	639 (559)	
Median (range)	250 (50-2250)	500 (75-3000)		200 (50-2250)	475 (75-2500)	
TRS status, No. (%)						
Complete	42 (81)	29 (83)	.60	34 (79%)	18 (82)	.64
Optimal	4 (8)	4 (11)		4 (9%)	3 (14)	
Suboptimal	6 (12)	2 (6)		5 (12%)	1 (5)	
Length of stay, d						
No.	52	35	.03	43	22	.08
Mean (SD)	6.1 (6.7)	7.8 (7.6)		6.4 (7.2)	6.8 (4.8)	
Median (range)	4.0 (0.0-34.0)	6.0 (2.0-43.0)		4.0 (0.0-34.0)	6.0 (2.0-25.0)	

Abbreviations: NA, not applicable; NACT, neoadjuvant chemotherapy; PIV, predictive index value; pTRS, primary tumor reductive surgery; TRS, tumor reductive surgery.

The frequencies of surgical procedures by morphologic subtype are described in eTable 2 in the Supplement. Compared with those with type I morphologic subtype, patients with predominant or uniform type II morphologic subtype were more likely to undergo a modified posterior exenteration (2% [1 of 57; 95% CI, 0%-9%] vs 19% [7 of 37; 95% CI, 8%-35%], *P* = .006; 2% [1 of 48; 95% CI, 0%-11%] vs 22% [5 of 23; 95% CI, 7%-44%], *P* = .01) or small bowel resection (2% [1 of 57; 95% CI, 0%-9%] vs 16% [6 of 37; 95% CI, 6%-32%], *P* = .01, 2% [1 of 48; 95% CI, 0%-11%] vs 22% [5 of 23; 95% CI, 7%-44%], *P* = .01). Patients with uniform type II morphologic subtype were also more likely to undergo peritoneal stripping (21% [10 of 48; 95% CI, 10%-35%] vs 48% [11 of 23; 95% CI, 27%-69%]; *P* = .02). Histopathologic review of 16 predominant type I cases and 12 predominant type II cases found that no microscopic histopathologic pattern dominated either subtype. However, papillary structures were identified in 50% of the type I specimens but only 8% of the type II specimens.

### Proteomic and Transcriptomic Analyses

**Figure 2.**
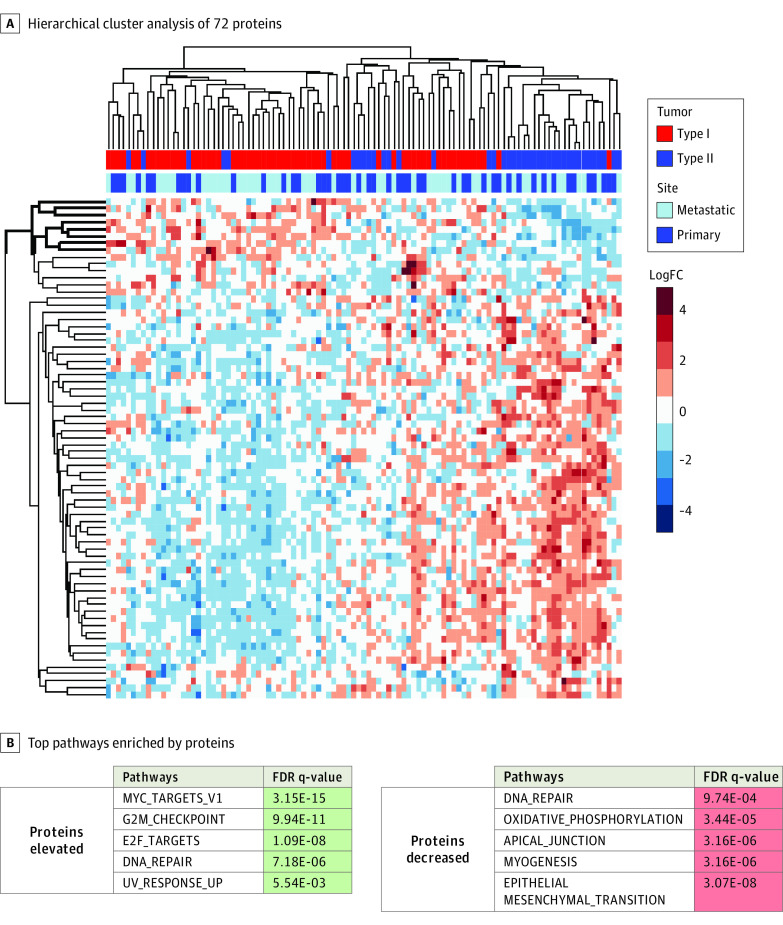
Differential Protein Expression Analysis of High-Grade Serous Ovarian Cancer (HGSOC) Type I and Type II Tumors A, Hierarchical cluster analysis of 72 proteins significantly altered between type II (n = 40) and type I (n = 63) HGSOC primary and metastatic tumors (linear models for microarray data [LIMMA]-adjusted *P* < .05). B, Top pathways enriched by proteins with significantly higher or lower expression in type II tumors than in type I tumors (LIMMA-adjusted *P* < .01). FC indicates fold change; FDR, false discovery rate.

To ascertain whether proteomic alterations were associated with either morphologic subtype, we used a highly multiplexed quantitative MS-based approach to quantify differential protein expression in primary and metastatic type I (n = 63) and type II (n = 40) tumors. Among the 5248 proteins analyzed, 72 had significantly different expression between type II and type I tumors (LIMMA-adjusted *P* < .05) (Figure 2A). Proteins with higher expression in type I tumors were significantly enriched in pathways involved in invasion and metastasis, including epithelial-mesenchymal transition (FDR q-value, 3.07 × 10^−8^; *P* < .001), hypoxia^13^ (FDR q-value, 2.05 × 10^−2^; *P* = .003), coagulation^14^ (FDR q-value, 4.31 × 10^−2^; *P* = .008), and glycolysis^15,16,17^ (FDR q-value, 2.05 × 10^−2^; *P* = .003) as well as the oxidative phosphorylation pathway (FDR q-value, 3.44 × 10^−5^; *P* < .001) and PI3K/AKT/mTOR (phosphoinositide 3-kinases/protein kinase B/mechanistic target of rapamycin) pathway (FDR q-value, 2.25 × 10^−2^; *P* = .004) (Figure 2B). In contrast, proteins with higher expression in type II tumors were significantly enriched in the MYC pathway (FDR q-value, 3.15 × 10^−15^; *P* < .001) and pathways involved in cell cycle progression (G2M checkpoint FDR q-value, 9.94 × 10^−11^; *P* < .001). Reverse phase protein array analysis similarly found that, compared with type I tumors, type II tumors had significantly higher expression of proteins involved in the positive regulation of the cell cycle and the FOXM1 transcription network^18,19,20^ (eTable 3 and eFigure 2 in the Supplement).

Transcriptomic analysis found a concordant increase in gene set enrichment analysis terms related to MYC signaling, proliferation, the cell cycle, and the FOXM1 transcription network in type II tumors (eFigure 3 and eFigure 4 in the Supplement). Also notable in transcriptomic-level data was the upregulation of immune pathways in type I compared with type II tumors. Specifically, the gene set enrichment analysis terms enriched in type I tumors included those related to the immunoglobulin complex, the T-cell receptor complex, phagocytosis recognition, and B-cell receptor activation and signaling.

Results from mRNA and protein profiling were highly concordant. Nearly 80% of all genes profiled by both methods (4093 of 5140) showed Spearman correlation coefficients greater than 0.2, with *P* < .05 when comparing protein and mRNA expression across 95 tumor samples (eFigure 5A in the Supplement). Furthermore, among the 820 proteins and/or transcripts that were differentially expressed between type I and type II tumors, there was a substantial correlation between mRNA-level and protein-level changes (Spearman ρ = 0.59; *P* < .001) (eFigure 5B in the Supplement).

Furthermore, we performed an integrated analysis of proteins and transcripts differentially expressed between type II tumors (n = 24) and type I (n = 37) tumors. We identified 201 protein-transcript pairs significantly co-altered between type II and type I tumors (LIMMA-adjusted *P* < .05) that further exhibited significant abundance correlation trends (Spearman ρ = 0.812; *P* < .001). Pathway analysis of these co-altered, protein-transcript pairs revealed that the pathways enriched in type II vs type I tumors were similar to those identified in the independent proteome and transcriptome analyses, including pathways involved in the activation of MYC targets (FDR q-value, 2.04 × 10^−9^; *P* < .001) and cell cycle regulatory signaling (G2M checkpoint FDR q-value, 1.10 × 10^−5^; *P* < .001). Moreover, the pathways enriched in type I vs type II tumors were similar to those identified in the independent proteome and transcriptome analyses, such as epithelial-mesenchymal transition (FDR q-value, 3.10 × 10^−24^; *P* < .001) and hypoxia (FDR q-value, 1.52 × 10^−5^; *P* < .001) pathways, but also included pathways involved in angiogenesis activation^21,22^ (FDR q-value, 2.11 × 10^−2^; *P* = .004) and hedgehog signaling^23,24^ (FDR q-value, 2.11 × 10^−2^; *P* = .004).

### Immune Profiling

We further investigated whether the infiltration of different immune cell populations was associated with morphologic subtype using immune-profiling analysis (eFigure 6 in the Supplement). Compared with predominant type II tissues, predominant type I tissues had significantly higher mean (SD) total T-cell infiltration in both the tumor area (2.45% [2.52%] vs 0.80% [0.94%]; *P* = .02) and total area (tumor and nontumor; 2.88% [2.69%] vs 1.06% [1.00%], *P* = .04). Specifically, predominant type I tissues had higher regulatory (Foxp3^+^) T-cell infiltration in the tumor area (0.73% [0.86%] vs 0.20% [0.19%]; *P* = .04), nontumor area (1.48% [1.48%] vs 0.37% [0.29%]; *P* = .03), and total area (0.89% [0.88%] vs 0.24% [0.19%]; *P* = .03). Predominant type I tumors had more helper (CD4^+^) T-cell infiltration than predominant type II tumors, but this difference was not significant. We did not find a difference in cytotoxic T-cell infiltration. The mean percentage of B-cell (CD20^+^) infiltration in type I tumors was 20 times that in type II tumors, but this difference was not statistically significant. Macrophage (CD68^+^ and CD163^+^) infiltration did not differ between type I and type II tumors. These results suggest that, compared with type II tumors, type I tumors have significantly higher infiltration of T cells, particularly regulatory T cells, and tend to have higher infiltration of helper T cells and B cells.

### Metabolomics Analysis

**Figure 3.**
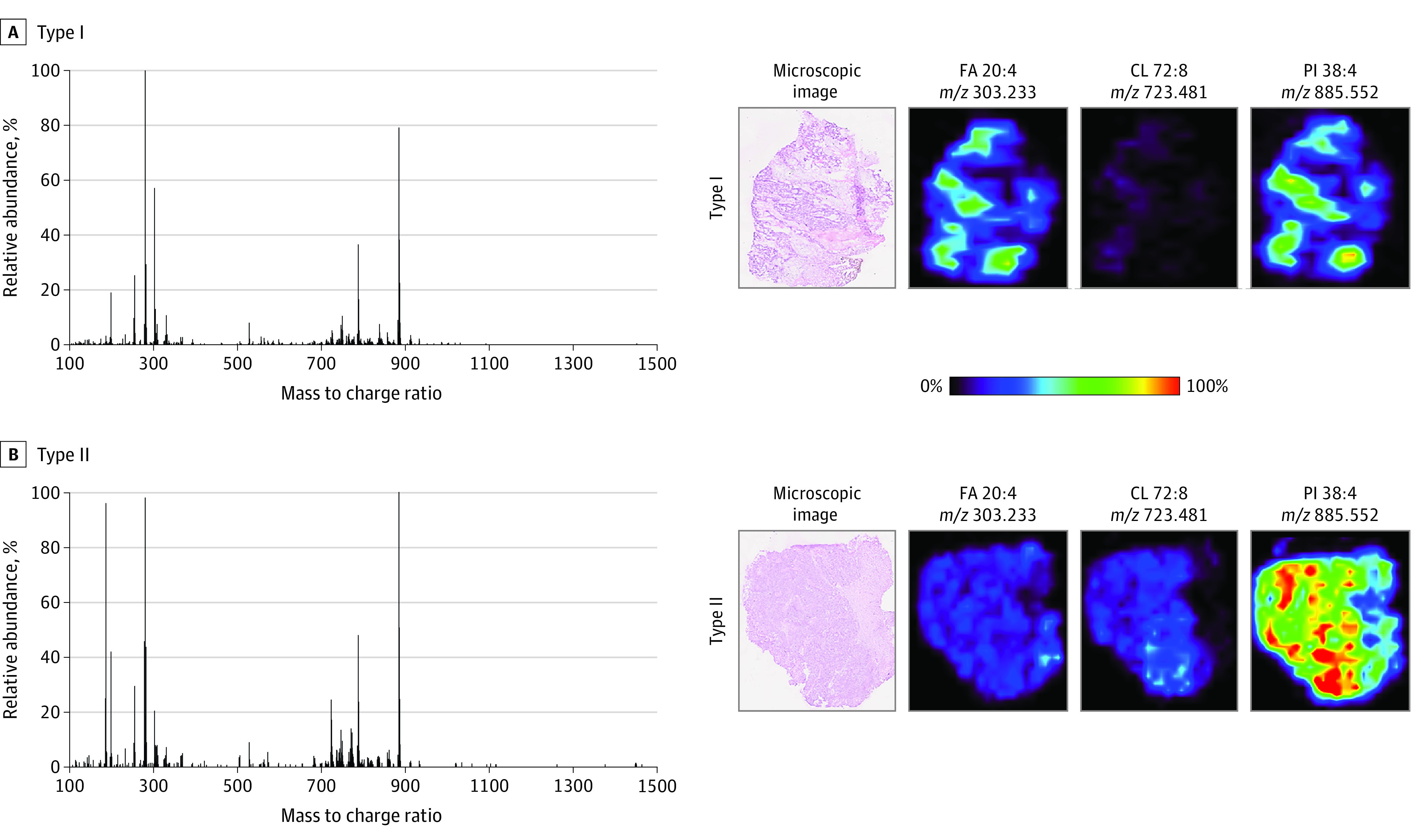
Representative Desorption Electrospray Ionization Mass Spectra and Ion Images for Primary Tissues of Type I and Type II Morphologic Subtypes In the ion images, red areas represent the highest relative abundances (100%), and black areas represent the lowest relative abundances (0%). Lipid species are described by their numbers of fatty acid chain carbons and double bonds. CL indicates cardiolipin; FA, fatty acid; PI, phosphatidylinositol.

Given the observation of higher expression of MYC pathways in type II tumors, and the known role of MYC in cancer cell metabolism,^25,26,27^ we used DESI-MS imaging to assess the in situ metabolomic profiles of tissue sections from 25 type I and 20 type II tumors. Representative MS and select ion images are shown in Figure 3 and eFigure 7 in the Supplement. The MS images obtained from tissues qualitatively presented similar metabolic profiles with subtle differences in the relative abundances of specific ions depending on morphologic subtype. Among primary tissues, type I tumors had a higher relative abundance of arachidonic acid, or fatty acid 20:4 (mass to charge ratio [*m/z*], 303.233), than type II tumors did, whereas type II tumors had higher relative abundances of cardiolipin 72:8 (*m/z*, 723.481) and phosphatidylinositol 38:4 (*m/z*, 885.552) than type I tumors did (Figure 3). Among metastatic tissues, type I tumors had a slightly higher relative abundance of phosphatidylserine 36:1 (*m/z*, 788.547), whereas type II tumors had higher relative abundances of oleic acid, or fatty acid 18:1 (*m/z*, 281.249), and phosphatidylglycerol 34:1 (*m/z*, 747.520) (eFigure 7 in the Supplement).

In primary tissues, SAM identified 140 ions in type I tumors and 101 ions in type II tumors with significantly higher relative abundances (eTable 4 and eTable 5 in the Supplement). In metastatic tissues, SAM identified 108 ions in type I tumors (eTable 6 in the Supplement) and 103 ions in type II tumors (eTable 7 in the Supplement) with significantly higher relative abundances. Most of the ions whose relative abundances were revealed by SAM to be significantly different between type I and II in both primary and metastatic tissues were tentatively identified as small metabolites (such as glucose, gluconic acid, glutathione, and benzoic acid) and as saturated and unsaturated fatty acids, glycerolipids, monoacylglycerophosphates, glycerophosphoethanolamines, glycerophosphocholines, glycerophosphoserines, glycerophosphoglycerols, glycerophosphoinositols, and cardiolipins. Collectively, these results suggest that the levels of certain metabolites and lipids differ substantially between type I and type II morphologic subtypes of HGSOC.

## Discussion

This study identifies a novel dichotomous categorization of the gross morphologic characteristics of HGSOC that have historically been observed at the time of tumor reductive surgery. Comparison of clinical outcomes and multiomic analysis identified key clinical and molecular differences between the 2 morphologic subtypes that could have implications for identifying outcomes, treatment planning, and developing novel targeted therapeutics.

Patients with type I and type II morphologic subtypes were found to have disparate surgical outcomes. Compared with those with type I, patients with type II were more likely to have a PIV lower than 8 and therefore were more often triaged to undergo primary tumor reductive surgery. These patients’ lower PIV scores may indicate that the exophytic appearance of type II lesions allows for aggressive cytoreduction, which may explain why patients with type II had longer operative times, higher estimated blood loss, and a higher likelihood of small bowel resection and/or exenteration than patients with type I.

To ascertain whether the observed differences in appearance and clinical outcomes may be associated with underlying molecular patterns, we performed multiomic analysis of tumor samples of each subtype. We found that compared with type II tumors, type I tumors exhibited enrichment of hedgehog signaling as well as signaling supporting epithelial-mesenchymal transition, hypoxia, angiogenesis, coagulation, and glycolysis, hallmarks of cancer associated with highly infiltrative lesions.^28,29,30,31,32^ In addition, given their increased hypoxia, which promotes the formation of chaotic, leaky, highly permeable vessels,^33^ and their increased epithelial-mesenchymal transition, which promotes cell mobility,^34^ type I tumors may have a predilection for hematogenous metastasis.

The findings also suggest that each morphologic subtype has potential therapeutic implications. The findings of the proteomic and transcriptomic pathway analyses suggest that type I morphologic subtype is enriched in PI3K/AKT/mTOR and hedgehog signaling, and therapies targeting each of these pathways are in development.^35,36^ Type I also had increased angiogenesis and thus may have a greater response to anti-angiogenic agents such as bevacizumab.^37^ Type II tumors exhibited increased MYC signaling. Type II tumors had a distinct lipid signature with significantly higher relative abundances of polyunsaturated phosphatidylglycerols and cardiolipin species compared with type I tissues, especially tissues from metastatic sites. Alterations in lipid signatures, including high abundances of phosphatidylglycerols and cardiolipins, have been associated with MYC as an oncogenic factor in lymphoma as well as renal and hepatocellular carcinoma,^38,39,40,41^ and c-MYC has been identified as a possible therapeutic target in platinum-resistant ovarian cancer.^42^ Thus, alterations in lipid signatures may help explain why patients with type II morphologic subtype tended to have a worse response to neoadjuvant chemotherapy, although this observation needs to be validated.

To investigate the tumor microenvironment of type I and type II tumors, we performed immune profiling. Type I had an increase in markers of immune infiltration, which suggests a potential opportunity to develop targeted therapy for HGSOC based on morphologic subtype. Further study is warranted to ascertain whether the morphologic subtype identified at the time of surgery could serve as a predictive marker of response to chemotherapy or immunotherapy.

These findings warrant validation in larger prospective studies. In addition, performing single cell–based analyses of tumor tissue would help elucidate the differences in the immune microenvironment and extracellular matrix between the morphologic subtypes. Studies investigating the extent to which either morphologic subtype is associated with specific radiographic findings should also be conducted to establish whether morphologic subtypes can be appreciated before entering the operating room. Furthermore, examining the degree of interrater concordance when teaching these novel categories or incorporating them into a standardized operative approach is important.

### Limitations

Because it involved a retrospective review of laparoscopic and clinical data, this study had inherent limitations. Misclassification bias was possible given the possibility of incomplete data in the medical record. In addition, selection bias may have altered treatment selection, and potential confounding variables may not have been fully assessed. Moreover, because this study was exploratory, an a priori power analysis was not performed; thus, the differences described with nonsignificant *P* values may have been significant, warranting additional prospective investigations.

## Conclusions

In this cohort study, we identified a novel, reliable means of categorizing HGSOC into 2 distinct subtypes based on gross morphologic appearance at the time of surgery. These subtypes’ unique molecular and metabolic signatures have possible implications for triaging patients to surgery or chemotherapy, identifying outcomes, and developing targeted therapeutics. These findings represent an original approach to establishing a systematic framework for classifying and managing patients on the basis of intraoperative morphologic observations.

## Notes

Supplement.
eMethods.
**eTable 1.** Baseline Patient Characteristics **eTable 2.** Frequency of Surgical Procedures **eTable 3.** Functional Annotations of Proteins With Higher Expression in Type II Than in Type I Tumors **eTable 4.** Tentative Attribution of Compounds Identified by SAM as Having Higher Relative Abundances in Primary Type I Than Type II Tumors **eTable 5.** Tentative Attribution of Compounds Identified by SAM as Having Higher Relative Abundances in Primary Type II Than Type I Tumors **eTable 6.** Tentative Attribution of Compounds Identified by SAM as Having Higher Relative Abundances in Metastatic Type I Than Type II Tumors **eTable 7.** Tentative Attribution of Compounds Identified by SAM as Having Higher Relative Abundances in Metastatic Type II Than Type I Tumors **eFigure 1.** Outline of the Study **eFigure 2.** NetWalker Analysis of Proteins With Significantly Altered Expression Levels Between Type I and Type II Tumors **eFigure 3.** Differential mRNA Transcript Expression Analysis of HGSOC Type I and Type II Tumors. Representative Gene Set Enrichment Analysis (GSEA) of RNA Sequencing Results Showing Pathway Changes in Primary (A) and Metastatic (B) Site Samples **eFigure 4.** Volcano Plots from RNA Sequencing of Patient-Derived (A) Primary and (B) Metastatic High-Grade Serous Ovarian Cancer Samples of Type I vs Type II Morphologic Subtypes **eFigure 5.** (A) Concordance Between All Genes and Corresponding Proteins Profiled by Both RNA Sequencing-Based Transcriptomics and Quantitative Mass Spectroscopy-Based Proteomics. (B) Correlation of Changes in Differentially Expressed Transcript/Protein Levels Between Type I and Type II Morphologic Subtypes **eFigure 6.** Immune Population Infiltration in Predominant Type I and Type II Morphologic Subtypes in the (A) Tumor Area and (B) Total Area (Tumor/Non-Tumor) **eFigure 7.** Representative Desorption Electrospray Ionization Mass Specta and Ion Images for Metastatic Tissues of Type I and Type II Morphologic Subtypes
eReferences
