# Supplementary material for: Classification of High-Grade Serous Ovarian Cancer Using Tumor Morphologic Characteristics
Source: JAMA Netw Open. 2022 Oct 14;5(10):e2236626. doi: 10.1001/jamanetworkopen.2022.36626 (PMC9568802; doi:10.1001/jamanetworkopen.2022.36626)
Supplement: Supplement. — eMethods. eTable 1. Baseline Patient Characteristics eTable 2. Frequency of Surgical Procedures eTable 3. Functional Annotations of Proteins With Higher Expression in Type II Than in Type I Tumors eTable 4. Tentative Attribution of Compounds Identified by SAM as Having Higher Relative Abundances in Primary Type I Than Type II Tumors eTable 5. Tentative Attribution of Compounds Identified by SAM as Having Higher Relative Abundances in Primary Type II Than Type I Tumors eTable 6. Tentative Attribution of Compounds Identified by SAM as Having Higher Relative Abundances in Metastatic Type I Than Type II Tumors eTable 7. Tentative Attribution of Compounds Identified by SAM as Having Higher Relative Abundances in Metastatic Type II Than Type I Tumors eFigure 1. Outline of the Study eFigure 2. NetWalker Analysis of Proteins With Significantly Altered Expression Levels Between Type I and Type II Tumors eFigure 3. Differential mRNA Transcript Expression Analysis of HGSOC Type I and Type II Tumors. Representative Gene Set Enrichment Analysis (GSEA) of RNA Sequencing Results Showing Pathway Changes in Primary (A) and Metastatic (B) Site Samples eFigure 4. Volcano Plots from RNA Sequencing of Patient-Derived (A) Primary and (B) Metastatic High-Grade Serous Ovarian Cancer Samples of Type I vs Type II Morphologic Subtypes eFigure 5. (A) Concordance Between All Genes and Corresponding Proteins Profiled by Both RNA Sequencing-Based Transcriptomics and Quantitative Mass Spectroscopy-Based Proteomics. (B) Correlation of Changes in Differentially Expressed Transcript/Protein Levels Between Type I and Type II Morphologic Subtypes eFigure 6. Immune Population Infiltration in Predominant Type I and Type II Morphologic Subtypes in the (A) Tumor Area and (B) Total Area (Tumor/Non-Tumor) eFigure 7. Representative Desorption Electrospray Ionization Mass Specta and Ion Images for Metastatic Tissues of Type I and Type II Morphologic Subtypes eReferences [file jamanetwopen-e2236626-s001.pdf]

Supplemental Online Content

Handley KF, Sims TT, Bateman NW, et al. Classification of high-grade serous ovarian cancer using tumor morphologic characteristics. *JAMA Netw Open*. 2022;5(10):e2236626. doi:10.1001/jamanetworkopen.2022.36626

eMethods.

eTable 1. Baseline Patient Characteristics

eTable 2. Frequency of Surgical Procedures

eTable 3. Functional Annotations of Proteins With Higher Expression in Type II Than in Type I Tumors

eTable 4. Tentative Attribution of Compounds Identified by SAM as Having Higher Relative Abundances in Primary Type I Than Type II Tumors

eTable 5. Tentative Attribution of Compounds Identified by SAM as Having Higher Relative Abundances in Primary Type II Than Type I Tumors

eTable 6. Tentative Attribution of Compounds Identified by SAM as Having Higher Relative Abundances in Metastatic Type I Than Type II Tumors

eTable 7. Tentative Attribution of Compounds Identified by SAM as Having Higher Relative Abundances in Metastatic Type II Than Type I Tumors

eFigure 1. Outline of the Study

eFigure 2. NetWalker Analysis of Proteins With Significantly Altered Expression Levels Between Type I and Type II Tumors

eFigure 3. Differential mRNA Transcript Expression Analysis of HGSOC Type I and Type II Tumors. Representative Gene Set Enrichment Analysis (GSEA) of RNA Sequencing Results Showing Pathway Changes in Primary (A) and Metastatic (B) Site Samples

eFigure 4. Volcano Plots from RNA Sequencing of Patient-Derived (A) Primary and (B) Metastatic High-Grade Serous Ovarian Cancer Samples of Type I vs Type II Morphologic Subtypes

© 2022 Handley KF et al. *JAMA Network Open.*

eFigure 5. (A) Concordance Between All Genes and Corresponding Proteins Profiled by Both RNA Sequencing-Based Transcriptomics and Quantitative Mass Spectroscopy-Based Proteomics. (B) Correlation of Changes in Differentially Expressed Transcript/Protein Levels Between Type I and Type II Morphologic Subtypes

eFigure 6. Immune Population Infiltration in Predominant Type I and Type II Morphologic Subtypes in the (A) Tumor Area and (B) Total Area (Tumor/Non-Tumor)

eFigure 7. Representative Desorption Electrospray Ionization Mass Specta and Ion Images for Metastatic Tissues of Type I and Type II Morphologic Subtypes

eReferences

This supplemental material has been provided by the authors to give readers additional information about their work.

© 2022 Handley KF et al. *JAMA Network Open.*

eMethods

Patient Selection and Clinical Analysis

This cohort study included patients who presented to MD Anderson with suspected advanced-stage epithelial ovarian cancer, underwent laparoscopic assessment of disease burden prior to treatment between April 1, 2013 and August 5, 2016, and received a histopathologic diagnosis of high-grade serous ovarian cancer (HGSOC). Patients excluded from the study were those who did not meet criteria for laparoscopic assessment of disease burden (e.g., distant metastatic or unresectable disease, co-morbidities precluding primary surgery, poor performance status) according to institutional consensus guidelines[1] as well as those who were not found to have HGSOC on final pathology and those without video recordings available. Patient data and laparoscopic videos were securely stored on the Research Electronic Data Capture software platform[2]. During laparoscopic video review, if both morphologic types were present in the same location, the site was classified as the predominating type. Response to NACT was classified as excellent if there was a complete response on radiologic evaluation according to Response Evaluation Criteria in Solid Tumors (RECIST 1.1), a normalization of CA-125 level (≤35 U/mL) in those whose baseline CA-125 level was at least twice the upper limit of normal, and/or a pathologic complete response. Response was classified as poor if there was progressive disease at cycle 1-4, stable disease at cycle 3-4 by RECIST 1.1, and/or suboptimal cytoreduction at interval tumor reductive surgery (iTRS). If a patient met one criteria for poor response and one criteria for excellent response, the patient was considered non-classifiable. All data collection and analysis was performed between April 2020 and November 2021.

Quantitative mass spectrometry-based Proteomics

Fifty-six frozen primary and metastatic tumor tissues from 32 patients diagnosed with HGSOC were thawed and analyzed by quantitative mass spectrometry (MS)-based proteomics as described previously[3]. Briefly, whole tumor (cancer and stromal cells) samples were harvested by laser microdissection, and samples were subjected to pressure-assisted digestion with trypsin employing a barocycler (2320EXT Pressure BioSciences, Inc). Ten micrograms of total peptide were labeled per tandem mass tag channel (TMTpro 16-plex, Thermo Fisher Scientific). Each multiplex included a reference channel, which was generated by pooling equivalent amounts of peptide digest from each of the 56 cancer specimens. The TMT multiplexes were resolved offline by basic reversed-phase liquid chromatography (1260 Infinity II liquid chromatograph, Agilent) into 24 pooled fractions. The pooled fractions were resuspended in 100 mM NH_4HCO_3 and analyzed by liquid chromatography (LC)-tandem MS (LC-MS/MS) employing a nanoflow LC system (EASY-nLC 1200, Thermo Fisher Scientific) coupled online with a Q-Exactive HF-X mass spectrometer (Thermo Fisher Scientific). Peptides were eluted by developing a linear gradient of 2% mobile phase B (95% acetonitrile, 0.1% formic acid) to 32% mobile phase B over 120 min at a constant flow rate of 250 nL/min. The electrospray source capillary voltage and temperature were set at 2.0 kV and 275°C, respectively. High-resolution (R=60,000 at *m/z* 200) broadband (*m/z* 400-1600) mass spectra were acquired, followed by selection of the top 12 most intense molecular ions in each MS scan for higher-energy collisional dissociation. Instrument-specific parameters were set as follows: broadband MS: Automatic Gain Control (AGC), 3e6; Radio Frequency Lens, 40%; Maximum Injection Time (IT), 45ms; Charge State, 2-4; Dynamic Exclusion, 10ppm/20 sec; MS2: AGC, 1e5; Maximum IT, 95ms; Resolution, 45k; Quadrupole Isolation, 1.0 *m/z*; Isolation Offset, 0.2 *m/z*; Normalized Collision Energy, 34; First Mass, 100; Intensity Threshold, 2e5; Tandem Mass Tag Optimization, On. Peptide and global protein-level identifications were generated by searching raw data files with a publicly available, non-redundant human proteome database (Swiss-Prot, http://www.uniprot.org/, downloaded 12-01-2017, 20257 entries) using Mascot (v2.6.0, Matrix Science), Proteome Discoverer (v2.2.0.388, Thermo Fisher Scientific), and in-house tools using identical parameters as described previously[3]. Global proteome level data were merged with global data for 47 frozen primary and metastatic tumors collected from 16 patients diagnosed with HGSOC described previously[3] and further classified as exhibiting type I or type II morphologic subtype followed by batch correction using the ComBat function in the R package sva (version 3.34.0).

Reverse Phase Protein Array (RPPA) and NetWalker Analysis

A total of 46 frozen tissues from 21 patients were prepared and analyzed by MD Anderson's Functional Proteomics RPPA Core Facility as described previously[3]. To validate the findings in patients with uniform morphologic subtype, we compared the median relative protein expression from the remaining patients' biopsy specimens classified as type I with those classified as type II, regardless of classification at the other sites.

© 2022 Handley KF et al. *JAMA Network Open.*

RNA Sequencing
Total RNA from 43 frozen tissues from 15 patients was prepared and sequenced as described previously[3]. Total RNA from another 64 frozen tissues from 32 patients were prepared by MD Anderson's Biospecimen Extraction Facility. In brief, RNA was extracted from frozen tissues using the Qiagen RNeasy kit (QIAGEN) according to the manufacturer's instructions. RNA qualification, RNA sequencing library generation, generation of sequencing data, and quality control proceeded as described previously, but with libraries sequenced on a Novaseq 6000 S4 platform[3]. FASTQ files from sequencing were mapped to human genome hg38 using Salmon software version 1.4.0 (https://combine-lab.github.io/salmon)[4] to produce raw counts and normalized transcripts per million (TPM) values. All analyses were performed using the R software functions and packages. Batch effects from two sequencing batches were removed using the ComBat function of the R sva package[5]. Differentially expressed genes were obtained using the DESeq2 package on raw counts with additional filtering by removing genes with low expression based on TPM. Tumor purities were estimated using the R ESTIMATE package[6]. GSEA was performed following the instructions from https://www.gsea-msigdb.org/gsea/index.jsp[7,8]. Pathway enrichment scores are calculated as the average of -log10(NOM p-value) and -log10(FDR q-value) reported by GSEA, which are ceiled at 9 and negatively signed for the control group.

Immune-profiling Analysis
Immune-profiling analysis was performed in tissues from patients with predominant type I morphologic subtype (n = 18, 8 primary and 10 metastatic sites) and patients with predominant type II morphologic subtype (n = 9, 3 primary and 6 metastatic sites) as previously described by Lee, et al[3]. In brief, Opal multiplex staining was performed on a single tissue section from each specimen. Multispectral imaging was utilized to obtain an average of five representative images per sample. Spectral unmixing was performed to separate raw images into individual fluorophores, which were analyzed to identify different cell types. Tumor vs. non-tumor areas in each sample were differentiated with a tissue segmentation algorithm.

MS Imaging
A total of 45 tissue sections were analyzed, of which 25 (14 primary and 11 metastases) were the type I morphologic subtype, and 20 (12 primary and 8 metastases) were the type II subtype. Glass slides containing 8-µm-thick tissue sections were stored in a freezer at -80ºC until use. Prior to desorption electrospray ionization (DESI)-MS analysis, the glass slides were dried for approximately 15 minutes. DESI-MS imaging was conducted as described previously[9]. An Omni Spray 2D Ion Source (Prosolia Inc.) equipped with a lab-built sprayer was coupled with a Q Exactive Focus Hybrid Quadrupole-Orbitrap mass spectrometer (Thermo Fisher Scientific). The analyses were performed in negative ion mode with a spatial resolution of 200 µm. A histologically compatible solvent system, dimethylformamide:acetonitrile (1:1 v/v), was used at a flow rate of 1.2 µL/min[10]. The N_2 pressure applied for the DESI sprayer was 185 psi. Mass spectra were collected over *m/z* 100-1500 with a resolving power of 70,000 at *m/z* 400. Other optimized instrumental parameters were as follows: spray voltage, 5 kV; capillary temperature, 300°C; S-lens radiofrequency level, 100; and an average of two micro-scans for each spectrum. The same tissue sections used for DESI-MS imaging were stained with hematoxylin and eosin. A board-certified pathologist with sub-specialty certification in gynecologic pathology (JL) performed pathologic evaluation using light microscopy to determine the tumor regions. Light microscopy images of hematoxylin and eosin–stained slides were obtained using the Nano Zoomer imaging system (Hamamatsu, model no. C13140-01).

© 2022 Handley KF et al. *JAMA Network Open.*

eTable 1. Baseline Patient Characteristics

Characteristic	Predominant Morphologic Subtype			Uniform Morphologic Subtype		
	Type I (n=57)	Type II (n=37)	p-value	Type I (n=48)	Type II (n=23)	p-value
Age at diagnosis, years			0.83			0.60
Mean (SD)	62.8 (9.0)	63.2 (10.0)		63.2 (9.1)	61.9 (11.1)	
Median (range)	62.0 (43.0-82.0)	64.0 (44.0-88.0)		62.5 (43.0-82.0)	62.0 (44.0-88.0)	
Race*, n (%)			0.45			0.60
American Indian or Alaskan Native	0 (0.0)	1 (2.9)		0 (0.0)	0(0.0)	
Asian	1 (1.8)	2 (5.7)		1 (2.1)	1 (4.5)	
Black or African American	6 (10.7)	3 (8.6)		5 (10.6)	3 (13.6)	
White or Caucasian	49 (87.5)	29 (82.9)		41 (87.2)	18 (81.8)	
Ethnicity*, n (%)			0.08			0.64
Hispanic or Latino	3 (5.4)	6 (17.1)		3 (6.4)	2 (9.5)	
Not Hispanic or Latino	53 (94.6)	29 (82.9)		44 (93.6)	19 (90.5)	
Preoperative BMI, kg/m^2			0.35			0.22
N	57	37		48	23	
Mean (SD)	27.1 (6.9)	28.3 (6.7)		26.9 (6.7)	28.8 (6.8)	
Median (range)	24.3 (18.0-47.2)	27.7 (17.1-41.7)		24.4 (18.0-47.2)	27.7 (19.6-41.4)	
Primary disease site, n (%)			0.90			>0.99
Fallopian tube	2 (3.5)	2 (5.4)		2 (4.2)	1 (4.3)	
Ovary	50 (87.7)	32 (86.5)		42(87.5)	20 (87.0)	
Peritoneum	5 (8.8)	3 (8.1)		4 (8.3)	2 (8.7)	
Stage, n (%)			0.94			0.72
IIB	1 (1.8)	0 (0.0)		1 (2.1)	0 (0.0)	
IIIC	49 (86.0)	31 (83.8)		41 (85.4)	18 (78.3)	
IVA	3 (5.3)	2 (5.4)		2 (4.2)	2 (8.7)	
IVB	4 (7.0)	4 (10.8)		4 (8.3)	3 (13.0)	
BRCA mutation status, n (%)			0.84			0.98
Patient declined testing	4 (8.2)	2 (6.9)		4 (10.0)	1 (4.8)	
No mutation	33 (67.4)	21 (72.4)		27 (67.5)	15 (71.4)	
BRCA1 mutated	6 (12.2)	4 (13.8)		5 (12.5)	3 (14.3)	
BRCA2 mutated	3 (6.1)	2 (6.9)		3 (7.5)	2 (9.5)	
VUS	3 (6.1)	0 (0.0)		1 (2.5)	0 (0.0)	
Baseline CA125, U/mL			0.14			0.21
N	56	37		48	23	
Median (range)	484.5 (11.5-6705.0)	620.4 (41.0-12472.0)		453.9 (11.5-6705.0)	469.5 (41.0-6882.0)	

© 2022 Handley KF et al. *JAMA Network Open.*

Characteristic (continued)	Predominant Morphologic Subtype			Uniform Morphologic Subtype		
	Type I (n=57)	Type II (n=37)	p-value	Type I (n=48)	Type II (n=23)	p-value
Platelets (x10^3/mm^3)			0.88			0.89
N	56	36		48	22	
Mean (SD)	386.3 (145.7)	373.9 (126.1)		386.2 (143.5)	381.4 (126.3)	
Median (range)	356.0 (144.0-928.0)	346.0 (160.0-733.0)		359.5 (167.0-928.0)	364.0 (160.0-653.0)	
Hemoglobin (g/dL)			0.20			0.29
N	53	36		46	23	
Mean (SD)	12.3 (1.4)	12.1 (1.2)		12.2 (1.5)	11.9 (1.3)	
Median (range)	12.7 (8.2-14.3)	12.3 (9.6-14.4)		12.6 (8.2-14.3)	12.1 (9.6-13.8)	
Hematocrit (%)			0.33			0.23
N	53	36		46	23	
Mean (SD)	37.7 (3.9)	37.2 (3.1)		37.7 (4.0)	36.8 (3.2)	
Median (range)	38.2 (27.2-43.5)	37.6 (31.6-43.1)		38.4 (27.2-43.5)	37.5 (31.6-42.9)	
Leukocytes (x10^3/mm^3)			0.15			0.11
N	53	36		46	23	
Mean (SD)	7.9 (2.2)	9.6 (6.2)		7.7 (2.2)	10.0 (7.5)	
Median (range)	7.6 (4.2-14.7)	8.5 (4.0-41.9)		7.5 (4.2-14.7)	8.8 (4.0-41.9)	
Neutrophils (x10^3/mm^3)			0.36			0.38
N	53	36		46	23	
Mean (SD)	5.5 (2.0)	6.1 (2.6)		5.4 (2.0)	6.0 (2.6)	
Median (range)	5.5 (1.2-12.3)	5.8 (1.8-13.8)		5.4 (1.2-12.3)	6.3 (1.8-10.7)	
Creatinine (mg/dL)			0.12			0.21
N	52	36		46	23	
Mean (SD)	0.8 (0.2)	0.8 (0.2)		0.8 (0.2)	0.8 (0.2)	
Median (range)	0.8 (0.5-1.4)	0.7 (0.4-1.2)		0.8 (0.5-1.4)	0.7 (0.4-1.2)	
Albumin (g/dL)			0.25			0.33
N	43	29		37	18	
Mean (SD)	4.0 (0.5)	3.8 (0.6)		4.0 (0.5)	3.8 (0.6)	
Median (range)	3.9 (2.5-5.0)	3.9 (2.5-4.8)		4.0 (2.5-5.0)	3.9 (2.5-4.6)	
ECOG Performance Status, n (%)			0.30			0.58
0	32 (57.1)	15 (40.5)		25 (53.2)	11 (47.8)	
1	20 (35.7)	18 (48.7)		18 (38.3)	8 (34.8)	
2	4 (7.1)	4 (10.8)		4 (8.5)	4 (17.4)	
ASA physical status class, n (%)			0.09			0.26
II	11 (19.6)	5 (13.9)		9 (19.2)	2 (9.1)	
III	45 (80.4)	28 (77.8)		38 (80.9)	19 (86.4)	
IV	0 (0.0)	3 (8.3)		0 (0.0)	1 (4.5)	

© 2022 Handley KF et al. *JAMA Network Open.*

Characteristic (continued)	Predominant Morphologic Subtype			Uniform Morphologic Subtype		
	**Type I (n=57)**	**Type II (n=37)**	**p-value**	**Type I (n=48)**	**Type II (n=23)**	**p-value**
Charlson Comorbidity Index, n (%)			0.38			0.91
N	57	37		48	23	
Mean (SD)	3.2 (1.7)	3.7 (2.4)		3.3 (1.7)	3.6 (2.6)	
Median (range)	3.0 (1.0-11.0)	3.0 (1.0-14.0)		3.0 (1.0-11.0)	3.0 (1.0-14.0)	
Employment status*, n (%)			0.06			0.23
Unemployed	29 (50.9%)	11 (29.7%)		24 (50.0%)	8 (34.8%)	
Employed part-time	1 (1.8%)	0 (0.0%)		1 (2.1%)	0 (0.0%)	
Employed full-time	14 (24.6%)	9 (24.3%)		13 (27.1%)	5 (21.7%)	
Unknown	13 (22.8%)	17 (46.0%)		10 (20.8%)	10 (43.5%)	

*Patient reported

Abbreviations: BMI, body mass index; VUS, variant of unknown significance; ECOG, Eastern Cooperative Oncology Group; ASA, American Society of Anesthesiologists

© 2022 Handley KF et al. *JAMA Network Open.*

eTable 2. Frequency of Surgical Procedures

Procedure	Predominant Morphologic Subtype			Uniform Morphologic Subtype		
	Type 1 (n=57), n (%)	Type 2 (n=37), n (%)	p-value	Type 1 (n=48), n (%)	Type 2 (n=23), n (%)	p-value
Hysterectomy			0.79			0.51
Not performed	14 (25)	10 (27)		14 (29)	5 (22)	
Performed	43 (75)	27 (73)		34 (71)	18 (78)	
Salpingo-oophorectomy			0.94			0.79
Not performed	5 (9)	2 (5)		5 (10)	1 (4)	
BSO	49 (86)	33 (89)		41 (85)	21 (91)	
RSO	2 (4)	1 (3)		1 (2)	0 (0)	
LSO	1 (2)	1 (3)		1 (2)	1 (4)	
Omentectomy			0.74			>0.99
Not performed	5 (9)	4 (11)		5 (10)	2 (9)	
Performed	52 (91)	33 (89)		43 (90)	21 (91)	
Argon beam coagulation			0.33			0.18
Not performed	32 (56)	17 (46)		29 (60)	10 (43)	
Performed	25 (44)	20 (54)		19 (40)	13 (57)	
Diaphragm procedures			0.33			0.82
Not performed	45 (79)	28 (76)		38 (79)	17 (74)	
Stripping	8 (14)	3 (8)		6 (13)	3 (13)	
Resection	4 (7)	6 (16)		4 (8)	3 (13)	
Modified posterior exenteration			0.006			0.01
Not performed	56 (98)	30 (81)		47 (98)	18 (78)	
Performed	1 (2)	7 (19)		1 (2)	5 (22)	
Liver resection			0.18			0.20
Not performed	53 (93)	31 (84)		45 (94)	19 (83)	
Performed	4 (7)	6 (16)		3 (6)	4 (17)	
Peritoneal stripping			0.13			0.02
Not performed	45 (79)	24 (65)		38 (79)	12 (52)	
Performed	12 (21)	13 (35)		10 (21)	11 (48)	
Splenectomy			0.66			>0.99
Not performed	51 (89)	32 (86)		42 (88)	20 (87)	
Performed	6 (11)	5 (14)		6 (13)	3 (13)	
Bladder resection			>0.99			>0.99
Not performed	56 (98)	37 (100)		47 (47)	23 (100)	
Performed	1 (2)	0 (0)		1 (2)	0 (0)	

© 2022 Handley KF et al. *JAMA Network Open.*

Procedure (continued)	Predominant Morphologic Subtype			Uniform Morphologic Subtype		
	Type 1 (n=57), n (%)	Type 2 (n=37), n (%)	p-value	Type 1 (n=48), n (%)	Type 2 (n=23), n (%)	p-value
Pelvic LND			>0.99			>0.99
Not performed	53 (93)	35 (95)		45 (94)	22 (96)	
Performed	4 (7)	2 (5)		3 (6)	1 (4)	
Para-aortic LND			>0.99			>0.99
Not performed	53 (93)	34 (92)		44 (92)	21 (91)	
Performed	4 (7)	3 (8)		4 (8)	2 (9)	
Appendectomy			0.29			0.43
Not performed	39 (68)	29 (78)		31 (65)	17 (74)	
Performed	18 (32)	8 (22)		17 (35)	6 (26)	
End colostomy			0.21			0.08
Not performed	55 (96)	33 (89)		46 (96)	19 (83)	
Performed	2 (4)	4 (11)		2 (4)	4 (17)	
Ileo-ascending resection			0.65			>0.99
Not performed	55 (96)	35 (95)		46 (96)	22 (96)	
Performed	2 (4)	2 (5)		2 (4)	1 (4)	
Loop ileostomy			0.21			>0.99
Not performed	55 (96)	33 (89)		46 (96)	22 (96)	
Performed	2 (4)	4 (11)		2 (4)	1 (4)	
Rectosigmoid resection			0.50			0.62
Not performed	45 (79)	27 (73)		38 (79)	17 (74)	
Performed	12 (21)	10 (27)		10 (21)	6 (26)	
Small bowel resection			0.01			0.01
Not performed	56 (98)	31 (84)		47 (98)	18 (78)	
Performed	1 (2)	6 (16)		1 (2)	5 (22)	
Resection of port site metastasis			0.15			0.30
Not performed	53 (93)	37 (100)		44 (92)	23 (100)	
Performed	4 (7)	0 (0)		4 (8)	0 (0)	

Abbreviations: BSO, bilateral salpingo-oophorectomy; RSO, right salpingo-oophorectomy; LSO, left salpingo-oophorectomy; LND, lymph node dissection

© 2022 Handley KF et al. *JAMA Network Open.*

eTable 3. Functional Annotations of Proteins With Higher Expression in Type II Than in Type I Tumors

Annotation ID	Functional Annotation	Ontology	Tree Level	Number of Occurrences	Fraction of Nodes	Hyper-geometric p-value	Genes
Bs119304	Progesterone-mediated oocyte maturation	Pathway	10	3	0.23	3.84E-08	*BRAF, PLK1, CCNB1*
Bs486750	Condensed nuclear chromosome	Structural complex	10	3	0.23	8.28E-09	*BRD4, PLK1, CCNB1*
Bs486751	Condensed chromosome	Structural complex	10	3	0.23	3.24E-07	*BRD4, PLK1, CCNB1*
Bs508122	Positive regulation of cell cycle	Pathway	10	3	0.23	6.51E-08	*BRD4, PLK1, CCNB1*
Bs137935	FOXM1 transcription network	Pathway	10	3	0.23	1.50E-09	*ESR1, PLK1, CCNB1*
Bs487083	Nuclear chromatin	Structural complex	10	3	0.23	2.90E-07	*BRD4, MUC1, ARID1A*
Bs494702	Positive regulation of organelle organization	Pathway	10	3	0.23	5.81E-07	*MUC1, PLK1, CCNB1*
Bs503	Progesterone-mediated oocyte maturation	Pathway	10	3	0.23	3.84E-08	*BRAF, PLK1, CCNB1*
Bs510652	Developmental growth	Pathway	10	3	0.23	8.52E-07	*ESR1, BRD4, CNB1*
Bs198811	Cell cycle	Pathway	10	3	0.23	4.82E-08	*CDH1, PLK1, CCNB1*
Bs198862	T cell receptor signaling pathway	Pathway	10	3	0.23	2.29E-07	*BRAF, MUC1, GAB2*
Bs518208	Positive regulation of cell cycle process	Pathway	10	3	0.23	3.24E-07	*BRD4, MUC1, PLK1*

© 2022 Handley KF et al. *JAMA Network Open.*

eTable 4. Tentative Attribution of Compounds Identified by SAM as Having Higher Relative Abundances in Primary Type I Than Type II Tumors

Tentative attribution	Molecular formula	Detected *m/z*	Mass error, ppm	SAM score
FA 8:0	$C_8H_{15}O_2$	143.1079	1.0	-22.738
FA 12:0	$C_{12}H_{23}O_2$	199.1701	-1.3	-17.936
FA 9:0	$C_9H_{17}O_2$	157.1227	-4.5	-17.575
FA 15:4	$C_{15}H_{21}O_2$	233.1547	0.0	-16.972
FA 18:0	$C_{18}H_{35}O_2$	283.2648	1.9	-13.502
FA 10:0	$C_{10}H_{19}O_2$	171.1385	-3.2	-12.432
PS 34:1	$C_{40}H_{75}NO_{10}P$	760.5149	2.0	-12.111
Uridine*	$C_9H_{11}N_2O_6$	243.0624	0.6	-11.371
PC 34:2	$C_{42}H_{80}NO_8PCl$	792.5314	-0.2	-10.724
Not identified	-	333.0946	-	-10.722
Not identified	-	188.0729	-	-10.208
Not identified	-	146.9650	-	-10.101
FA 20:4	$C_{20}H_{31}O_2$	303.2333	1.1	-10.018
Cer d42:1	$C_{42}H_{83}NO_3Cl$	684.6072	0.7	-9.940
Not identified	-	186.0492	-	-9.631
FA 16:0	$C_{16}H_{31}O_2$	255.2333	1.4	-9.582
LacCer d18:0/26:0*	$C_{56}H_{108}NO_{13}$	1002.7896	7.0	-8.971
Not identified	-	114.9351	-	-8.967
Not identified	-	368.9771	-	-8.725
FA 14:0	$C_{14}H_{27}O_2$	227.2014	-1.1	-8.564
PE 34:2	$C_{39}H_{73}NO_8P$	714.5109	4.2	-8.105
PE 44:11*	$C_{49}H_{75}NO_8P$	836.5314	9.4	-7.836
PG 32:0	$C_{38}H_{75}O_{10}P$	721.5026	0.1	-7.787
PE O-34:3 or PE P-34:2	$C_{39}H_{73}NO_7P$	698.5133	0.4	-7.530
PA 24:2	$C_{37}H_{68}O_8P$	671.4676	2.8	-7.408
PI 32:0	$C_{41}H_{78}O_{13}P$	809.5214	3.5	-7.268
PE 34:1	$C_{39}H_{75}NO_8P$	716.5248	1.7	-7.100
PE 36:3	$C_{41}H_{75}NO_8P$	740.5232	-0.5	-6.999
PI 38:2	$C_{47}H_{86}O_{13}P$	889.5768	-4.9	-6.716
PI 36:1	$C_{45}H_{84}O_{13}P$	863.5643	-1.4	-6.361
Not identified	-	191.0726	-	-6.283
Not identified	-	126.9040	-	-6.260
LysoPI 18:0	$C_{27}H_{52}O_{12}P$	599.3215	2.2	-6.182
FA 22:0	$C_{22}H_{43}O_2$	339.3269	0.1	-6.091
Cer d38:1	$C_{40}H_{79}NO_3Cl$	656.5768	2.1	-6.039
Not identified	-	116.9334	-	-5.957
PA 36:4	$C_{39}H_{68}O_8P$	695.4690	4.7	-5.810
PA 32:0	$C_{35}H_{68}O_8P$	647.4686	4.4	-5.691

© 2022 Handley KF et al. *JAMA Network Open.*

Tentative attribution (continued)	Molecular formula	Detected *m/z*	Mass error, ppm	SAM score
CL 72:4	$C_{81}H_{148}O_{17}P_2$	727.5077	-3.3	-5.405
PE 36:1	$C_{41}H_{79}NO_8P$	744.5533	-2.1	-5.399
Not identified	-	348.1582	-	-5.144
FA 13:8	$C_{13}H_9O_2$	197.0605	-1.5	-5.124
FA 20:0	$C_{20}H_{39}O_2$	311.2956	0.1	-5.089
DG 38:4/0:0	$C_{41}H_{72}O_5Cl$	679.5090	2.4	-4.971
FA 20:5	$C_{20}H_{29}O_2$	301.2174	0.3	-4.914
PI 36:1	$C_{45}H_{84}O_{13}P$	863.5679	2.8	-4.885
PE 36:2	$C_{41}H_{77}NO_8P$	742.5407	2.0	-4.694
Cer d18:16:0	$C_{34}H_{69}NO_3Cl$	574.4982	1.8	-4.679
PE 39:5	$C_{44}H_{77}NO_8P$	778.5414	2.8	-4.598
Mannitol*	$C_6H_{13}O_6$	181.0710	-4.2	-4.425
Not identified	-	356.2645	-	-4.314
2-Dodecylbenzenesulfonic acid*	$C_{18}H_{29}O_3S$	325.1852	2.8	-4.265
Not identified	-	412.9677	-	-4.256
CL 74:10	$C_{83}H_{140}O_{17}P_2$	735.4814	3.5	-4.159
PI 34:2	$C_{43}H_{78}O_{13}P$	833.5211	3.1	-4.158
CL 72:6	$C_{81}H_{144}O_{17}P_2$	725.4940	-0.7	-4.103
PE O-40:6*	$C_{45}H_{79}NO_7P$	776.5604	0.6	-4.061
PE 18:1/1:0	$C_{24}H_{45}NO_8P$	506.2899	2.1	-3.995
Cer m42:1	$C_{42}H_{83}NO_2Cl$	668.6140	3.3	-3.987
PG 22:1	$C_{38}H_{72}O_{10}P$	719.4879	1.4	-3.970
PE 38:3	$C_{43}H_{79}NO_8P$	768.5546	-0.4	-3.842
Not identified	-	160.9109	-	-3.720
Not identified	-	158.9109	-	-3.693
Not identified	-	265.1472	-	-3.632
PG 36:2	$C_{42}H_{78}O_{10}P$	773.5358	2.6	-3.578
PC 36:2	$C_{44}H_{84}NO_8PCl$	820.5603	-3.1	-3.527
Benzoic acid	$C_7H_5O_2$	121.0290	-4.2	-3.509
FA 11:0	$C_{11}H_{21}O_2$	185.1541	-3.3	-3.416
Glutamic acid	$C_5H_8NO_4$	146.0449	-6.7	-3.413
PE 38:5	$C_{43}H_{75}NO_8P$	764.5244	1.1	-3.277
PI 38:6	$C_{47}H_{78}O_{13}P$	881.5209	2.7	-3.208
PA 36:3	$C_{39}H_{70}O_8P$	697.4815	0.2	-3.140
N-Undecanoylglycine*	$C_{13}H_{24}NO_3$	242.1764	1.0	-3.116
Prolyl-Glutamine*	$C_{10}H_{17}N_3O_4Cl$	278.0929	5.7	-2.960
Not identified	-	698.9738	-	-2.929
PS 36:2	$C_{42}H_{77}NO_{10}P$	786.5322	4.0	-2.902
PE O-34:2 or PE P-34:1	$C_{39}H_{75}NO_7P$	700.5309	3.2	-2.834
PI 38:5	$C_{47}H_{80}O_{13}P$	883.5338	-0.5	-2.744
Cer d18:1/20:0*	$C_{38}H_{75}NO_3Cl$	628.5462	3.3	-2.737

© 2022 Handley KF et al. *JAMA Network Open.*

Tentative attribution (continued)	Molecular formula	Detected *m/z*	Mass error, ppm	SAM score
PG dO-40:9*	$C_{46}H_{76}O_8P$	787.5334	6.4	-2.734
MG 20:4*	$C_{23}H_{38}O_4Cl$	413.2465	0.2	-2.679
Glucose	$C_6H_{12}O_6Cl$	215.0327	-0.4	-2.655
PE O-38:5	$C_{43}H_{77}NO_7P$	750.5443	0.0	-2.643
PS O-36:2 or PS P-36:1	$C_{42}H_{79}NO_9P$	772.5517	2.5	-2.623
3-Hydroxypicolinic acid	$C_6H_4NO_3$	138.0188	-6.3	-2.539
Not identified	-	123.9012	-	-2.531
Cer d18:2/28:0*	$C_{46}H_{89}NO_3Cl$	738.6605	9.3	-2.483
Methymycin	$C_{25}H_{43}NO_7Cl$	504.2725	-1.7	-2.465
PE O-36:2*	$C_{41}H_{79}NO_7P$	728.5631	4.3	-2.456
Valerenolic acid	$C_{15}H_{21}O_3$	249.1494	-0.9	-2.431
FA 24:1	$C_{24}H_{45}O_2$	365.3427	0.5	-2.411
PG 36:4	$C_{42}H_{74}O_{10}P$	769.5059	4.4	-2.402
Not identified	-	130.9303	-	-2.390
CL 68:5	$C_{77}H_{138}O_{17}P_2$	698.4730	2.8	-2.327
PG 40:8	$C_{46}H_{74}O_{10}P$	817.5011	1.7	-2.278
FA 10:6;O*	$C_{10}H_7O_3$	175.0409	4.8	-2.193
CL 70:4	$C_{79}H_{144}O_{17}P_2$	713.4935	-1.4	-2.106
FA 24:0	$C_{24}H_{47}O_2$	367.3586	1.2	-2.084
PS 38:3	$C_{44}H_{79}NO_{10}P$	812.5447	0.0	-2.081
FAHFA 36:1;O*	$C_{36}H_{69}O_4$	565.5208	1.2	-2.078
Not identified	-	910.5576	-	-2.033
PE 39:1	$C_{44}H_{86}NO_8PCl$	822.5730	-6.7	-2.015
FA 17:0	$C_{17}H_{33}O_2$	269.2489	1.1	-1.985
Cer d40:2	$C_{40}H_{77}NO_3Cl$	654.5615	2.7	-1.984
FA 12:2	$C_{12}H_{19}O_2$	195.1387	-1.8	-1.955
PG 38:5	$C_{44}H_{76}O_{10}P$	795.5153	-3.6	-1.921
FA 22:1	$C_{22}H_{41}O_2$	337.3115	0.9	-1.917
PG 38:6	$C_{44}H_{74}O_{10}P$	793.5020	-0.6	-1.900
PS P-36:2 or PS O-36:3	$C_{42}H_{77}NO_9P$	770.5349	1.0	-1.895
PS 42:6*	$C_{48}H_{82}O_{10}NP$	862.5556	-5.5	-1.811
CL 78:6*	$C_{87}H_{156}O_{17}P_2$	767.5438	3.1	-1.803
PE O-36:3 or P-36:2	$C_{41}H_{77}NO_7P$	726.5459	2.2	-1.745
Gluconic acid	$C_6H_{11}O_7$	195.0505	-2.7	-1.657
Histidine*	$C_6H_8N_3O_2$	154.0613	-5.8	-1.641
PS 42:2	$C_{48}H_{89}O_{10}NP$	870.6248	2.1	-1.619
PE 39:3*	$C_{44}H_{82}NO_8PCl$	818.5497	3.0	-1.611
PI 25:1	$C_{44}H_{82}O_{13}P$	849.5519	-2.4	-1.579
PE 41:6*	$C_{46}H_{79}NO_8P$	804.5570	2.6	-1.397
PA 24:1	$C_{37}H_{70}O_8P$	673.4832	2.7	-1.365
PI O-23:0	$C_{42}H_{83}O_{12}PCl$	845.5325	1.0	-1.360

© 2022 Handley KF et al. *JAMA Network Open.*

Tentative attribution (continued)	Molecular formula	Detected *m/z*	Mass error, ppm	SAM score
PS 36:3	$C_{42}H_{75}NO_{10}P$	784.5139	0.6	-1.299
PE 0:0/20:4;O2*	$C_{25}H_{43}NO_9P$	532.2698	3.2	-1.252
PE 39:4*	$C_{44}H_{80}NO_8PCl$	816.5395	9.7	-1.248
MG 20:0	$C_{23}H_{46}O_4Cl$	421.3103	3.1	-1.245
PG 40:7	$C_{46}H_{76}O_{10}P$	819.5203	2.6	-1.229
Not identified	-	369.9810	-	-1.166
PG 40:5	$C_{46}H_{80}O_{10}P$	823.5496	0.2	-1.110
Not identified	-	421.3319	-	-1.084
PE O-36:4*	$C_{41}H_{75}NO_7P$	724.5296	1.3	-1.064
PS 37:2*	$C_{43}H_{79}NO_{10}P$	800.5501	6.7	-1.061
PI 36:3	$C_{45}H_{80}O_{13}P$	859.5347	0.6	-1.059
Not identified	-	614.3848	-	-1.055
PI 40:5	$C_{49}H_{84}O_{13}P$	911.5638	-1.9	-1.046
PI 36:3*	$C_{45}H_{81}O_{13}P$	859.5352	1.2	-0.931
DG 34:2/0:0	$C_{37}H_{68}O_5Cl$	627.4758	-0.4	-0.875
Cer d34:2	$C_{34}H_{65}NO_3Cl$	570.4672	2.4	-0.868
PI O-32:0*	$C_{41}H_{80}O_{12}P$	795.5396	0.4	-0.851
PE O-40:5 or PE P-40:4	$C_{45}H_{81}NO_7P$	778.5781	3.2	-0.838
PS O-38:4 or P-38:3	$C_{44}H_{79}NO_9P$	796.5483	-1.9	-0.808
PS 37:1*	$C_{43}H_{81}NO_{10}P$	802.5659	6.9	-0.802

Data are ranked based on SAM score values (highest to lowest). Attributions were assigned based on high mass accuracy and MS/MS measurements. Ions identified based on only high mass accuracy are marked with asterisks.

Abbreviations: SAM, significance analysis of microarrays; FA, fatty acid; PS, phosphatidylserine; PC, phosphatidylcholine; Cer, ceramide; LacCer, lactosylceramide; PE, phosphatidylethanolamine; PG, phosphatidylglycerol; PA, phosphatidic acid; PI, phosphatidylinositol; LysoPI, lysophosphatidylinositol; CL, cardiolipin; DG, diglyceride; MG, monoglyceride; FAHFA, fatty acid ester of hydroxyl fatty acid

© 2022 Handley KF et al. *JAMA Network Open.*

eTable 5. Tentative Attribution of Compounds Identified by SAM as Having Higher Relative Abundances in Primary Type II Than Type I Tumors

Tentative attribution	Molecular formula	Detected *m/z*	Mass error, ppm	SAM score
Not identified	-	186.0449	-	23.579
PS 44:8	$C_{50}H_{81}O_{10}NP$	886.5554	-5.6	16.018
Not identified	-	289.0371	-	14.279
Not identified	-	736.9918	-	12.787
FA 24:4	$C_{24}H_{39}O_2$	359.2959	1.0	12.049
FA 26:5	$C_{26}H_{41}O_2$	385.3105	1.8	12.036
CL 74:9	$C_{83}H_{142}O_{17}P_2$	736.4893	3.6	11.842
FA 18:3	$C_{18}H_{29}O_2$	277.2178	1.8	11.407
Glutathione	$C_{10}H_{16}N_3O_6S$	306.0773	2.5	11.139
FA 24:5	$C_{24}H_{37}O_2$	357.2807	2.2	10.641
Not identified	-	115.0195	-	10.476
PS 40:5	$C_{46}H_{79}NO_{10}P$	836.5367	-9.6	10.448
PS 40:4<br>N-acetylaspartic acid	$C_{46}H_{81}NO_{10}P$<br>$C_6H_8NO_5$	838.5644<br>174.0402	4.8<br>-3.4	10.303<br>10.271
Not identified	-	737.9970	-	10.002
FA 26:4	$C_{26}H_{43}O_2$	387.3260	2.3	9.984
Not identified	-	750.0001	-	9.774
PE O-38:6*	$C_{43}H_{75}NO_7P$	748.5255	-4.2	8.814
Not identified	-	142.9978	-	8.742
MG 18:0	$C_{21}H_{40}O_4Cl$	391.2620	-0.2	8.668
PE 40:5	$C_{45}H_{79}NO_8P$	792.5582	4.2	8.609
FA 20:3	$C_{20}H_{33}O_2$	305.2491	1.6	8.584
PS 38:2	$C_{44}H_{81}NO_{10}P$	814.5577	3.3	8.505
Not identified	-	556.3059	-	8.274
PS O-38:6*	$C_{44}H_{75}NO_9P$	792.5223	4.8	8.028
PE O-36:5*	$C_{41}H_{73}NO_7P$	722.5150	2.8	8.017
PS 38:4	$C_{44}H_{77}NO_{10}P$	810.5296	0.7	7.983
PE 20:4/1:0	$C_{26}H_{43}NO_8P$	528.2728	-0.8	7.766
CL 72:8	$C_{81}H_{140}O_{17}P_2$	723.4808	2.7	7.759
FA 17:1	$C_{17}H_{31}O_2$	267.2335	2.0	7.693
FA 22:5	$C_{22}H_{33}O_2$	329.2494	2.4	7.674
PS 40:3	$C_{46}H_{83}O_{10}NP$	842.5744	-1.9	7.606
PG 42:4*	$C_{48}H_{87}O_{10}PCl$	889.5720	-1.2	7.559
FA 18:1	$C_{18}H_{32}O_2$	281.2492	2.1	7.128
PA O-38:2 or PA P-38:1	$C_{41}H_{79}O_7PCl$	749.5278	1.3	6.959
PG 34:1	$C_{40}H_{76}O_{10}P$	747.5204	3.0	6.880
PI 39:4	$C_{48}H_{85}O_{13}PCl$	935.5458	3.9	6.809
FA 18:2	$C_{18}H_{31}O_2$	279.2327	1.1	6.706

© 2022 Handley KF et al. *JAMA Network Open.*

Tentative attribution (continued)	Molecular formula	Detected *m/z*	Mass error, ppm	SAM score
Cer d14:1/24:1*	$C_{38}H_{73}NO_3Cl$	626.5350	10.0	6.544
Xanthine*	$C_5H_3N_4O_2$	151.0255	-4.3	6.521
CL 74:8	$C_{83}H_{144}O_{17}P_2$	737.4921	3.3	6.376
FA 16:1	$C_{16}H_{29}O_2$	253.2177	1.6	6.343
PG 36:3	$C_{42}H_{76}O_{10}P$	771.5201	-2.5	6.340
PE O-38:7*	$C_{43}H_{73}NO_7P$	746.5132	0.3	6.154
PS 36:1	$C_{42}H_{79}NO_{10}P$	788.5466	-2.4	6.035
PE 39:6	$C_{44}H_{75}NO_8P$	776.5258	2.9	6.001
Taurine	$C_2H_6NO_3S$	124.0064	-8.0	5.874
PE O-38:3*	$C_{43}H_{82}NO_7PCl$	790.5535	1.5	5.748
FA 22:4	$C_{22}H_{35}O_2$	331.2649	2.0	5.710
PE P-18:0/18:4*	$C_{41}H_{73}NO_7Cl$	722.5116	1.9	5.625
PS O-33:0*	$C_{39}H_{78}NO_9P$	770.5071	-4.9	5.597
PI 38:4	$C_{47}H_{82}O_{13}P$	885.5522	2.7	5.591
FA 26:2	$C_{26}H_{47}O_2$	391.3587	1.4	5.333
PS 44:9	$C_{50}H_{79}O_{10}NP$	884.5385	-7.0	5.171
PI 38:3	$C_{47}H_{84}O_{13}P$	887.5629	-2.9	5.127
FA 22:2	$C_{22}H_{39}O_2$	335.2952	1.2	5.049
CL 78:8	$C_{87}H_{152}O_{17}P_2$	765.5271	1.7	4.957
PE O-38:4*	$C_{43}H_{79}NO_7P$	752.5554	-6.1	4.851
FA 26:0	$C_{26}H_{51}O_2$	395.3889	1.5	4.808
FA 20:2	$C_{20}H_{35}O_2$	307.2638	-1.6	4.658
FA 24:2	$C_{24}H_{43}O_2$	363.3262	1.9	4.619
PS 35:2*	$C_{41}H_{75}NO_{10}P$	772.5183	6.3	4.571
PI 34:1	$C_{43}H_{80}O_{13}P$	835.5342	0.0	4.535
PE 37:5	$C_{42}H_{73}NO_8P$	750.5078	-0.1	4.527
PS 39:4*	$C_{45}H_{79}NO_{10}P$	824.5454	0.8	4.405
FA 26:1	$C_{26}H_{49}O_5$	393.3734	1.0	4.340
FA 22:3	$C_{22}H_{37}O_2$	333.2794	1.5	4.303
PS P-34:1*	$C_{40}H_{75}NO_9P$	744.5231	6.2	4.114
PE 38:6	$C_{43}H_{73}NO_8P$	762.5082	0.4	3.958
FA 19:1	$C_{19}H_{35}O_2$	295.2644	0.5	3.949
CL 72:7	$C_{81}H_{142}O_{17}P_2$	724.4841	3.6	3.704
PA 36:1	$C_{39}H_{74}O_8P$	701.5120	1.0	3.702
PI 37:4	$C_{46}H_{80}O_{13}P$	871.5358	1.8	3.675
CL 74:7	$C_{83}H_{146}O_{17}P_2$	738.5015	1.1	3.582
Cer d18:2/24:1*	$C_{42}H_{79}NO_3Cl$	680.5770	2.4	3.224
PG 40:6	$C_{46}H_{78}O_{10}P$	821.5309	3.5	3.202
PI 40:5	$C_{49}H_{84}O_{13}P$	911.5667	1.3	3.076
PG 38:3	$C_{44}H_{80}O_{10}P$	799.5467	3.5	2.886
Pyroglutamate	$C_5H_6NO_3$	128.0343	-7.9	2.779

© 2022 Handley KF et al. *JAMA Network Open.*

Tentative attribution (continued)	Molecular formula	Detected *m/z*	Mass error, ppm	SAM score
PS 40:2	$C_{46}H_{85}O_{10}NP$	842.5906	1.3	2.739
Cer d34:1	$C_{34}H_{67}NO_3Cl$	572.4818	0.5	2.717
PS 42:1	$C_{48}H_{91}O_{10}NP$	872.6408	2.5	2.497
FA 24:3	$C_{24}H_{41}O_2$	361.3106	1.7	2.401
PG 18:0/18:0	$C_{42}H_{82}O_{10}P$	777.5651	0.3	2.383
PE 40:4	$C_{45}H_{81}NO_8P$	794.5711	0.7	2.322
PI 40:4	$C_{49}H_{86}O_{13}P$	913.5793	2.1	2.043
FA 22:6	$C_{22}H_{31}O_2$	327.2326	1.2	2.005
LysoPG 18:1	$C_{24}H_{46}O_9P$	509.2881	-2.9	1.889
PG 38:4	$C_{44}H_{78}O_{10}P$	797.5313	3.1	1.701
FA 15:0	$C_{15}H_{29}O_2$	241.2172	-0.4	1.582
CL 76:8	$C_{85}H_{148}O_{17}P_2$	751.5086	2.0	1.451
DG 34:1/0:0	$C_{37}H_{70}O_5Cl$	629.4926	1.4	1.379
MG 18:2*	$C_{21}H_{38}O_4Cl$	389.2478	-3.3	1.206
Cer d36:1	$C_{36}H_{71}NO_3Cl$	600.5143	2.5	1.134
PG 18:0/18:1	$C_{42}H_{80}O_{10}P$	775.5507	1.6	1.087
PG 42:9	$C_{48}H_{76}O_{10}P$	843.5198	1.9	1.081
DG 36:2/0:0	$C_{39}H_{72}O_5Cl$	655.5080	1.0	1.077
FA 20:1	$C_{20}H_{37}O_2$	309.2795	2.3	1.014
PE 38:4	$C_{43}H_{77}NO_8P$	766.5411	2.4	0.992
Not identified	-	1104.7753	-	0.962
PI P-18:0/17:2	$C_{44}H_{81}O_{12}PCl$	867.5187	3.2	0.846

Data are ranked based on SAM score values (highest to lowest). Attributions were assigned based on high mass accuracy and MS/MS measurements. Ions identified based on only high mass accuracy are marked with asterisks.

Abbreviations: SAM, significance analysis of microarrays; FA, fatty acid; CL, cardiolipin; PE, phosphatidylethanolamine; MG, monoglyceride; PG, phosphatidylglycerol; PA, phosphatidic acid; PI, phosphatidylinositol; Cer, ceramide; LysoPG, lysophosphatidylglycerol; DG, diglyceride; MG, monoglyceride

© 2022 Handley KF et al. *JAMA Network Open.*

eTable 6. Tentative Attribution of Compounds Identified by SAM as Having Higher Relative Abundances in Metastatic Type I Than Type II Tumors

Tentative attribution	Molecular formula	Detected *m/z*	Mass error, ppm	SAM score
FA 12:0	$C_{12}H_{23}O_2$	199.1700	-1.8	-27.359
PI 36:1	$C_{45}H_{84}O_{13}P$	863.5660	0.6	-22.054
FA 9:0	$C_9H_{17}O_2$	157.1226	-5.1	-21.089
PI 32:0	$C_{41}H_{78}O_{13}P$	809.5214	3.5	-19.010
PI 34:1	$C_{43}H_{80}O_{13}P$	835.5368	3.1	-18.869
FA 8:0	$C_8H_{15}O_2$	143.1068	-6.7	-17.678
PI 34:0	$C_{43}H_{82}O_{13}P$	837.5465	-4.0	-16.978
PS 40:4	$C_{46}H_{81}NO_{10}P$	838.5624	2.4	-16.454
FA 10:0	$C_{10}H_{19}O_2$	171.1385	-3.2	-15.064
PE O-38:5	$C_{43}H_{77}NO_7P$	750.5452	1.2	-14.994
Gluconic acid	$C_6H_{11}O_7$	195.0506	-2.2	-14.851
Uridine*	$C_9H_{11}N_2O_6$	243.0624	0.6	-13.261
PI 36:2	$C_{45}H_{82}O_{13}P$	861.5486	1.5	-12.448
PE O-40:5 or PE P-40:4	$C_{45}H_{81}NO_7P$	778.5781	3.2	-11.774
Cer d34:1	$C_{34}H_{67}NO_3Cl$	572.4819	0.7	-11.681
Cer d42:0	$C_{42}H_{81}NO_3Cl$	682.5906	-0.7	-11.212
Not identified	-	126.9040	-	-11.100
PS 38:4	$C_{44}H_{77}NO_{10}P$	810.5308	1.9	-10.963
Cer d42:1	$C_{42}H_{83}NO_3Cl$	684.6072	0.7	-10.917
PS 42:6*	$C_{48}H_{82}O_{10}NP$	862.5556	-5.5	-10.473
Cer d36:1	$C_{36}H_{71}NO_3Cl$	600.5143	2.5	-10.214
Cer d38:1	$C_{40}H_{79}NO_3Cl$	656.5779	3.8	-9.935
PI 38:2	$C_{47}H_{86}O_{13}P$	889.5752	-6.7	-9.714
PS 36:1	$C_{42}H_{79}NO_{10}P$	788.5466	-2.4	-9.425
FA 15:4	$C_{15}H_{21}O_2$	233.1546	-0.4	-9.386
PI 40:5	$C_{49}H_{84}O_{13}P$	911.5638	-1.9	-9.078
Not identified	-	713.9970	-	-8.686
PA 36:1	$C_{39}H_{74}O_8P$	701.5134	1.0	-8.538
Not identified	-	146.9650	-	-8.533
PE O-38:3*	$C_{43}H_{82}NO_7PCl$	790.5528	0.6	-8.070
Not identified	-	186.0492	-	-7.868
PE O-40:8	$C_{45}H_{76}NO_8PCl$	808.5068	1.8	-7.761
PS 36:2	$C_{42}H_{77}NO_{10}P$	786.5322	4.0	-7.456
CL 72:4	$C_{81}H_{148}O_{17}P_2$	727.5054	2.0	-7.263
Not identified	-	333.0946	-	-7.173
PE O-34:2 or PE P-34:1	$C_{39}H_{75}NO_7P$	700.5309	3.2	-7.100
PS 40:3	$C_{46}H_{83}NO_{10}P$	840.5746	-1.7	-7.023
FA 11:0	$C_{11}H_{21}O_2$	185.1541	-3.3	-6.346

© 2022 Handley KF et al. *JAMA Network Open.*

Tentative attribution (continued)	Molecular formula	Detected *m/z*	Mass error, ppm	SAM score
PE O-38:6*	$C_{43}H_{75}NO_7P$	748.5279	2.3	-6.337
LysoPI 18:0	$C_{27}H_{52}O_{12}P$	599.3215	2.2	-6.335
PI 32:1	$C_{41}H_{76}O_{13}P$	807.5016	1.6	-6.104
PI 25:1	$C_{44}H_{82}O_{13}P$	849.5519	-2.4	-6.022
Not identified	-	188.0729	-	-5.991
CL 70:4	$C_{79}H_{144}O_{17}P_2$	713.4935	-1.4	-5.880
PE 39:5	$C_{44}H_{77}NO_8P$	778.5414	2.8	-5.601
FA 7:0	$C_7H_{13}O_2$	129.0915	-4.7	-5.576
PA 24:1	$C_{37}H_{70}O_8P$	673.4832	2.7	-5.495
PI 34:2	$C_{43}H_{78}O_{13}P$	833.5211	3.1	-5.466
PS 42:4*	$C_{48}H_{85}NO_{10}P$	866.5924	0.9	-5.370
PE 18:1/1:0	$C_{24}H_{45}NO_8P$	506.2899	2.1	-5.243
PS 40:2	$C_{46}H_{85}O_{10}NP$	842.5906	1.3	-5.066
PS 38:1	$C_{44}H_{83}O_{10}NP$	816.5745	1.8	-5.065
PE O-36:2*	$C_{41}H_{79}NO_7P$	728.5619	2.6	-4.998
Not identified	-	191.0726	-	-4.893
PE O-36:3 or P-36:2	$C_{41}H_{77}NO_7P$	726.5459	2.2	-4.655
PE 39:1	$C_{44}H_{86}NO_8PCl$	822.5730	-6.7	-4.541
PS O-40:5*	$C_{46}H_{81}O_9NP$	822.5660	0.7	-4.541
FA 15:0	$C_{15}H_{29}O_2$	241.2172	-0.4	-4.388
PG dO-40:9*	$C_{46}H_{76}O_8P$	787.5333	6.4	-4.294
PS 38:3	$C_{44}H_{79}NO_{10}P$	812.5463	2.0	-4.242
PS 38:2	$C_{44}H_{81}NO_{10}P$	814.5577	3.3	-4.193
CL 80:8	$C_{89}H_{156}O_{17}P_2$	779.5440	3.3	-4.020
PS 34:1	$C_{40}H_{75}NO_{10}P$	760.5158	3.1	-2.917
FA 10:6;O*	$C_{10}H_7O_3$	175.0409	4.8	-2.852
Cer m42:1	$C_{42}H_{83}NO_2Cl$	668.6140	3.3	-2.824
Glucose	$C_6H_{12}O_6Cl$	215.0326	-0.9	-2.749
PE 36:1	$C_{41}H_{79}NO_8P$	744.5558	1.2	-2.728
PS 40:1	$C_{46}H_{87}O_{10}NP$	844.6080	0.8	-2.708
Not identified	-	265.1472	-	-2.684
PS 42:2	$C_{48}H_{89}O_{10}NP$	870.6248	2.1	-2.554
FA 17:0	$C_{17}H_{33}O_2$	269.2489	1.1	-2.486
Benzoic acid	$C_7H_5O_2$	121.0286	-7.5	-2.484
Not identified	-	293.1762	-	-2.430
FA 20:5	$C_{20}H_{29}O_2$	301.2174	0.3	-2.428
PI 40:4	$C_{49}H_{86}O_{13}P$	913.5816	0.5	-2.274
PS 44:8	$C_{50}H_{81}O_{10}NP$	886.5557	-5.2	-2.199
MG 20:0	$C_{23}H_{46}O_4Cl$	421.3103	3.1	-2.133
Prolyl-Glutamine*	$C_{10}H_{17}N_3O_4Cl$	278.0929	5.7	-2.102
PA 32:0	$C_{35}H_{68}O_8P$	647.4686	4.4	-2.071

© 2022 Handley KF et al. *JAMA Network Open.*

Tentative attribution (continued)	Molecular formula	Detected *m/z*	Mass error, ppm	SAM score
Not identified	-	556.3044	-	-1.841
PS 37:4*	$C_{43}H_{75}NO_{10}P$	796.5167	4.1	-1.777
Not identified	-	146.9820	-	-1.638
PE 39:4*	$C_{44}H_{80}NO_{8}PCl$	816.5395	9.7	-1.610
PG 42:3*	$C_{48}H_{89}O_{10}PCl$	891.5912	2.8	-1.609
2-Dodecylbenzenesulfonic acid*	$C_{18}H_{29}O_{3}S$	325.1850	2.3	-1.605
FA 13:8	$C_{13}H_{9}O_{2}$	197.0605	-1.5	-1.589
FA 18:0	$C_{18}H_{35}O_{2}$	283.2648	1.9	-1.494
Not identified	-	348.1582	-	-1.490
Valerenolic acid	$C_{15}H_{21}O_{3}$	249.1495	-0.5	-1.469
Not identified	-	152.9853	-	-1.464
PG 40:8	$C_{46}H_{74}O_{10}P$	817.5011	1.7	-1.211
Capryloylglycine*	$C_{10}H_{19}NO_{3}Cl$	236.1057	-0.8	-1.082
PG 44:5	$C_{50}H_{89}O_{10}PCl$	915.5917	3.2	-1.055
MG 18:2*	$C_{21}H_{38}O_{4}Cl$	389.2478	-3.3	-1.037
DG 34:1/0:0	$C_{37}H_{70}O_{5}Cl$	629.4917	0.0	-0.943
Not identified	-	699.9829	-	-0.815
PG 40:5	$C_{46}H_{80}O_{10}P$	823.5496	0.2	-0.745
MG 18:0	$C_{21}H_{40}O_{4}Cl$	391.2620	-0.2	-0.620
PE O-36:5*	$C_{41}H_{73}NO_{7}P$	722.5158	3.9	-0.619
Cer d18:1/20:0*	$C_{38}H_{75}NO_{3}Cl$	628.5458	2.7	-0.559
PA 40:1	$C_{43}H_{83}O_{8}PCl$	793.5601	10.3	-0.551
PE 34:1	$C_{39}H_{75}NO_{8}P$	716.5255	2.7	-0.534
Not identified	-	114.9351	-	-0.490
FA 12:5;O2*	$C_{12}H_{13}O_{4}$	221.0815	-2.0	-0.432
Not identified	-	289.0371	-	-0.362
PE O-38:4*	$C_{43}H_{79}NO_{7}P$	752.5563	-4.9	-0.294
PE 28:2;O3*	$C_{33}H_{61}NO_{11}P$	678.3983	-0.7	-0.101
Xanthine*	$C_{5}H_{3}N_{4}O_{2}$	151.0252	-6.3	-0.099

Data are ranked based on SAM score values (highest to lowest). Attributions were assigned based on high mass accuracy and MS/MS measurements. Ions identified based on only high mass accuracy are marked with asterisks.

Abbreviations: SAM, significance analysis of microarrays; FA, fatty acid; PI, phosphatidylinositol; PS, phosphatidylserine; PE, phosphatidylethanolamine; Cer, ceramide; PA, phosphatidic acid; CL, cardiolipin; LysoPI, lysophosphatidylinositol; PG, phosphatidylglycerol; MG, monoglyceride; DG, diglyceride

© 2022 Handley KF et al. *JAMA Network Open.*

eTable 7. Tentative Attribution of Compounds Identified by SAM as Having Higher Relative Abundances in Metastatic Type II Than Type I Tumors

Tentative attribution	Molecular formula	Detected *m/z*	Mass error, ppm	SAM score
PG 34:1	$C_{40}H_{76}O_{10}P$	747.5198	2.2	22.505
FA 18:2	$C_{18}H_{31}O_2$	279.2335	2.0	21.522
FA 18:1	$C_{18}H_{32}O_2$	281.2492	2.1	17.735
N-acetylaspartic acid	$C_6H_8NO_5$	174.0402	-3.4	17.214
CL 72:8	$C_{81}H_{140}O_{17}P_2$	723.4811	3.1	16.476
PG 36:4	$C_{42}H_{74}O_{10}P$	769.5059	4.4	14.636
PG 34:2	$C_{40}H_{74}O_{10}P$	745.5015	-1.4	14.597
Taurine	$C_2H_6NO_3S$	124.0064	-8.0	13.866
PG 36:3	$C_{42}H_{76}O_{10}P$	771.5201	-2.5	12.985
PG 36:2	$C_{42}H_{78}O_{10}P$	773.5340	0.2	12.916
FA 20:1	$C_{20}H_{37}O_2$	309.2795	2.3	12.742
CL 74:9	$C_{83}H_{142}O_{17}P_2$	736.4906	5.3	12.670
FA 20:2	$C_{20}H_{35}O_2$	307.2638	-1.6	12.546
Ascorbic acid	$C_6H_7O_6$	175.0243	-2.9	12.545
PS 35:2*	$C_{41}H_{75}NO_{10}P$	772.5187	6.8	12.516
Not identified	-	736.9918	-	12.069
CL 70:7	$C_{79}H_{138}O_{17}P_2$	710.4689	3.0	11.960
Glutamine	$C_5H_9N_2O_3$	145.0621	-1.4	11.747
PA O-38:2 or PA P-38:1	$C_{41}H_{79}O_7PCl$	749.5278	1.3	11.508
PE O-38:7*	$C_{43}H_{73}NO_7P$	746.5132	0.3	10.513
CL 74:8	$C_{83}H_{144}O_{17}P_2$	737.4921	3.3	10.300
CL 72:7	$C_{81}H_{142}O_{17}P_2$	724.4855	-1.6	10.259
Glutathione	$C_{10}H_{16}N_3O_6S$	306.0771	1.9	9.958
FA 22:5	$C_{22}H_{33}O_2$	329.2493	2.1	9.806
PS P-36:2 or PS O-36:3	$C_{42}H_{77}NO_9P$	770.5368	3.4	9.770
PG 40:7	$C_{46}H_{76}O_{10}P$	819.5203	2.6	9.134
FA 22:4	$C_{22}H_{35}O_2$	331.2649	2.0	9.122
PS O-36:4 or PS P-36:3	$C_{42}H_{75}NO_9P$	768.5211	-3.4	9.054
PI 36:4	$C_{45}H_{78}O_{13}P$	857.5210	2.9	8.847
CL 78:8	$C_{87}H_{152}O_{17}P_2$	765.5271	1.7	8.741
PG 38:3	$C_{44}H_{80}O_{10}P$	799.5467	3.5	8.607
CL 74:6	$C_{83}H_{148}O_{17}P_2$	739.5074	3.7	8.205
FA 16:1	$C_{16}H_{29}O_2$	253.2176	1.2	8.076
FA 22:2	$C_{22}H_{39}O_2$	335.2966	3.1	7.838
FA 18:3	$C_{18}H_{29}O_2$	277.2178	1.8	7.782
FA 20:3	$C_{20}H_{33}O_2$	305.2493	1.3	7.619
PG 32:0	$C_{38}H_{75}O_{10}P$	721.5026	0.1	7.618
PE 36:3	$C_{41}H_{75}NO_8P$	740.5263	3.7	7.549

© 2022 Handley KF et al. *JAMA Network Open.*

Tentative attribution (continued)	Molecular formula	Detected *m/z*	Mass error, ppm	SAM score
PE 36:2	$C_{41}H_{77}NO_8P$	742.5407	2.0	7.477
PI 38:4	$C_{47}H_{82}O_{13}P$	885.5522	2.7	7.192
Cer d18:2/28:0*	$C_{46}H_{89}NO_3Cl$	738.6605	9.3	7.059
PG 18:0/18:1	$C_{42}H_{80}O_{10}P$	775.5507	1.6	6.738
PG 40:6	$C_{46}H_{78}O_{10}P$	821.5309	3.5	6.593
PE 20:3/1:0*	$C_{26}H_{45}NO_8P$	530.2912	-4.5	6.542
FA 19:1	$C_{19}H_{35}O_2$	295.2644	0.5	6.337
FA 26:2	$C_{26}H_{47}O_2$	391.3587	1.4	6.329
PE 38:5	$C_{43}H_{75}NO_8P$	764.5244	1.1	6.223
Not identified	-	116.9334	-	6.024
Not identified	-	158.9109	-	5.940
FA 22:1	$C_{22}H_{41}O_2$	337.3115	0.9	5.875
Not identified	-	160.9109	-	5.822
PS 37:2*	$C_{43}H_{79}NO_{10}P$	800.5501	6.7	5.717
PS 37:1*	$C_{43}H_{81}NO_{10}P$	802.5659	6.9	5.716
FA 16:0	$C_{16}H_{31}O_2$	255.2333	1.4	5.492
CL 76:8	$C_{85}H_{148}O_{17}P_2$	751.5086	2.0	5.450
CL 70:6	$C_{79}H_{140}O_{17}P_2$	711.4767	3.0	5.339
PE 38:6	$C_{43}H_{73}NO_8P$	762.5082	0.4	5.063
Not identified	-	115.0195	-	4.884
2-Dodecylbenzenesulfonic acid*	$C_{18}H_{29}O_3S$	325.1850	2.3	4.761
3-Hydroxypicolinic acid	$C_6H_4NO_3$	138.0187	-7.0	4.740
FA 24:2	$C_{24}H_{43}O_2$	363.3262	1.9	4.619
PE 20:4/1:0	$C_{26}H_{43}NO_8P$	528.2728	-0.8	4.585
PS O-38:4 or P-38:3	$C_{44}H_{79}NO_9P$	796.5483	-1.9	4.547
CL 72:6	$C_{81}H_{144}O_{17}P_2$	725.4940	-0.7	4.451
FA 20:0	$C_{20}H_{39}O_2$	311.2956	0.1	4.437
PA 36:3	$C_{39}H_{70}O_8P$	697.4815	0.2	4.426
PS 44:9	$C_{50}H_{79}O_{10}NP$	884.5385	-7.0	4.401
PI 38:5	$C_{47}H_{80}O_{13}P$	883.5338	-0.5	4.396
PI 37:4	$C_{46}H_{80}O_{13}P$	871.5358	1.8	4.386
PG 38:4	$C_{44}H_{78}O_{10}P$	797.5313	3.1	4.316
PI 37:3	$C_{46}H_{82}O_{13}P$	873.5506	-0.8	4.234
FA 22:3	$C_{22}H_{37}O_2$	333.2794	1.5	4.210
FA 24:4	$C_{24}H_{39}O_2$	359.2959	1.0	3.968
PE O-36:4*	$C_{41}H_{75}NO_7P$	724.5296	1.3	3.851
FA 22:6	$C_{22}H_{31}O_2$	327.2333	1.1	3.720
PI 38:3	$C_{47}H_{84}O_{13}P$	887.5629	-2.9	3.366
PE 34:2	$C_{39}H_{73}NO_8P$	714.5109	4.2	3.350
PE-Cer d37:1	$C_{39}H_{79}N_2O_6PCl$	737.5359	1.5	3.252
FA 20:4*	$C_{20}H_{32}O_2Cl$	339.2088	2.4	3.242

© 2022 Handley KF et al. *JAMA Network Open.*

Tentative attribution (continued)	Molecular formula	Detected *m/z*	Mass error, ppm	SAM score
LysoPI 15:0	$C_{24}H_{46}O_{12}P$	557.2729	0.5	3.161
Glutamic acid	$C_5H_8NO_4$	146.0449	-6.7	3.130
Pyroglutamate	$C_5H_6NO_3$	128.0343	-7.9	3.071
PE 37:5	$C_{42}H_{73}NO_8P$	750.5078	-0.1	3.071
FA 26:1	$C_{26}H_{49}O_5$	393.3734	1.0	3.011
FA 14:0	$C_{14}H_{27}O_2$	227.2014	-1.1	2.912
PG 22:1	$C_{38}H_{72}O_{10}P$	719.4879	1.4	2.891
PS 42:1	$C_{48}H_{91}O_{10}NP$	872.6408	2.5	2.856
PG 38:6	$C_{44}H_{74}O_{10}P$	793.5020	-0.6	2.411
CL 79:7	$C_{85}H_{150}O_{17}P_2$	752.5180	0.1	2.236
PI 38:6	$C_{47}H_{78}O_{13}P$	881.5209	2.7	2.182
PG 18:0/18:0	$C_{42}H_{82}O_{10}P$	777.5651	0.3	2.150
CL 78:6*	$C_{87}H_{156}O_{17}P_2$	767.5438	3.1	2.082
FA 26:0	$C_{26}H_{51}O_2$	395.3889	1.5	1.982
Not identified	-	356.2645	-	1.827
DG 36:3/0:0	$C_{39}H_{70}O_5Cl$	653.4928	1.6	1.734
Not identified	-	123.9012	-	1.709
FA 24:1	$C_{24}H_{45}O_2$	365.3427	0.5	1.618
FA 17:1	$C_{17}H_{31}O_2$	267.2331	0.5	1.417
PA 24:2	$C_{37}H_{68}O_8P$	671.4676	2.8	1.324
CL 74:0*	$C_{83}H_{160}O_{17}P_2$	745.5604	4.4	1.287
PS 39:3	$C_{45}H_{81}NO_{10}P$	826.5628	-2.9	1.195
FA 9:1;O2*	$C_9H_{16}O_4$	187.0972	-2.0	1.150
PE 39:6	$C_{44}H_{75}NO_8P$	776.5258	2.9	1.122

Data are ranked based on SAM score values (highest to lowest). Attributions were assigned based on high mass accuracy and MS/MS measurements. Ions identified based on only high mass accuracy are marked with asterisks.
Abbreviations: SAM, significance analysis of microarray; PG, phosphatidylglycerol; FA, fatty acid; CL, cardiolipin; PS, phosphatidylserine; PA, phosphatidic acid; PE, phosphatidylethanolamine; PI, phosphatidylinositol; Cer, ceramide; LysoPI, lysophosphatidylinositol; DG, diglyceride

© 2022 Handley KF et al. *JAMA Network Open.*

eFigure 1. Outline of the Study

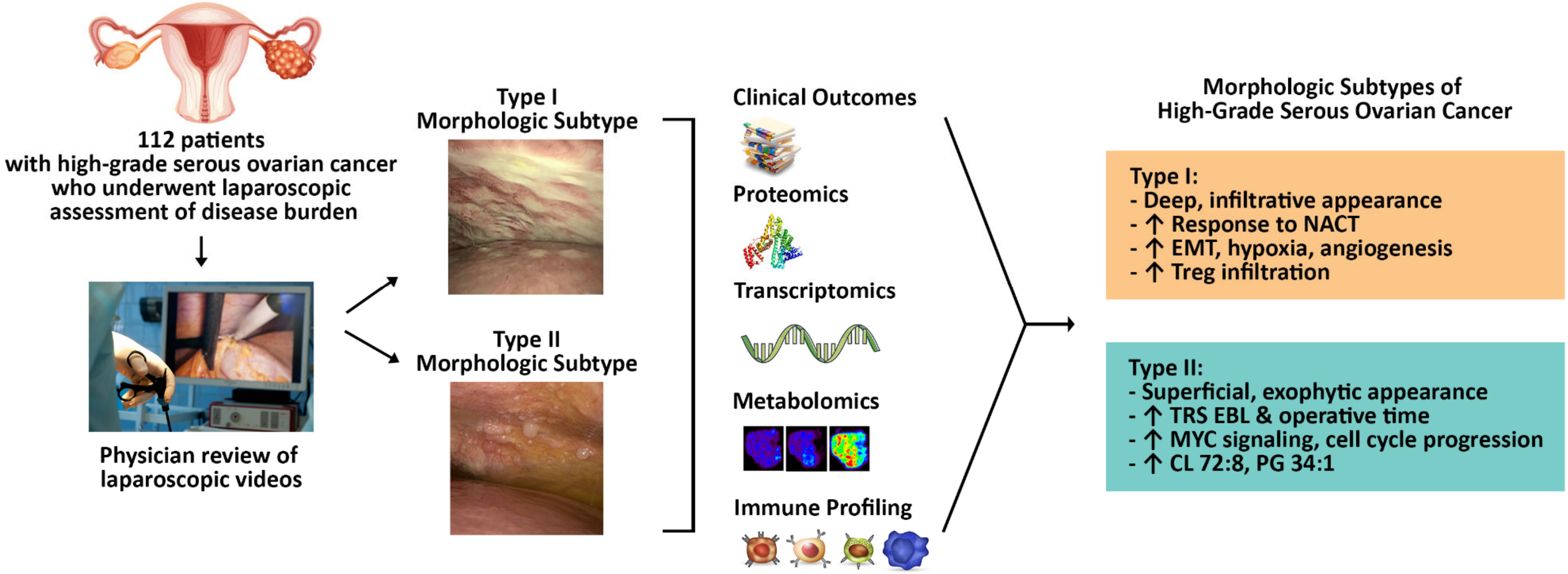

Abbreviations: NACT, neoadjuvant chemotherapy; EMT, epithelial-mesenchymal transition; Treg, regulatory T cell; TRS, tumor reductive surgery; CL, cardiolipin; PG, phosphatidylglycerol

© 2022 Handley KF et al. *JAMA Network Open.*

eFigure 2. NetWalker Analysis of Proteins With Significantly Altered Expression Levels Between Type I and Type II Tumors

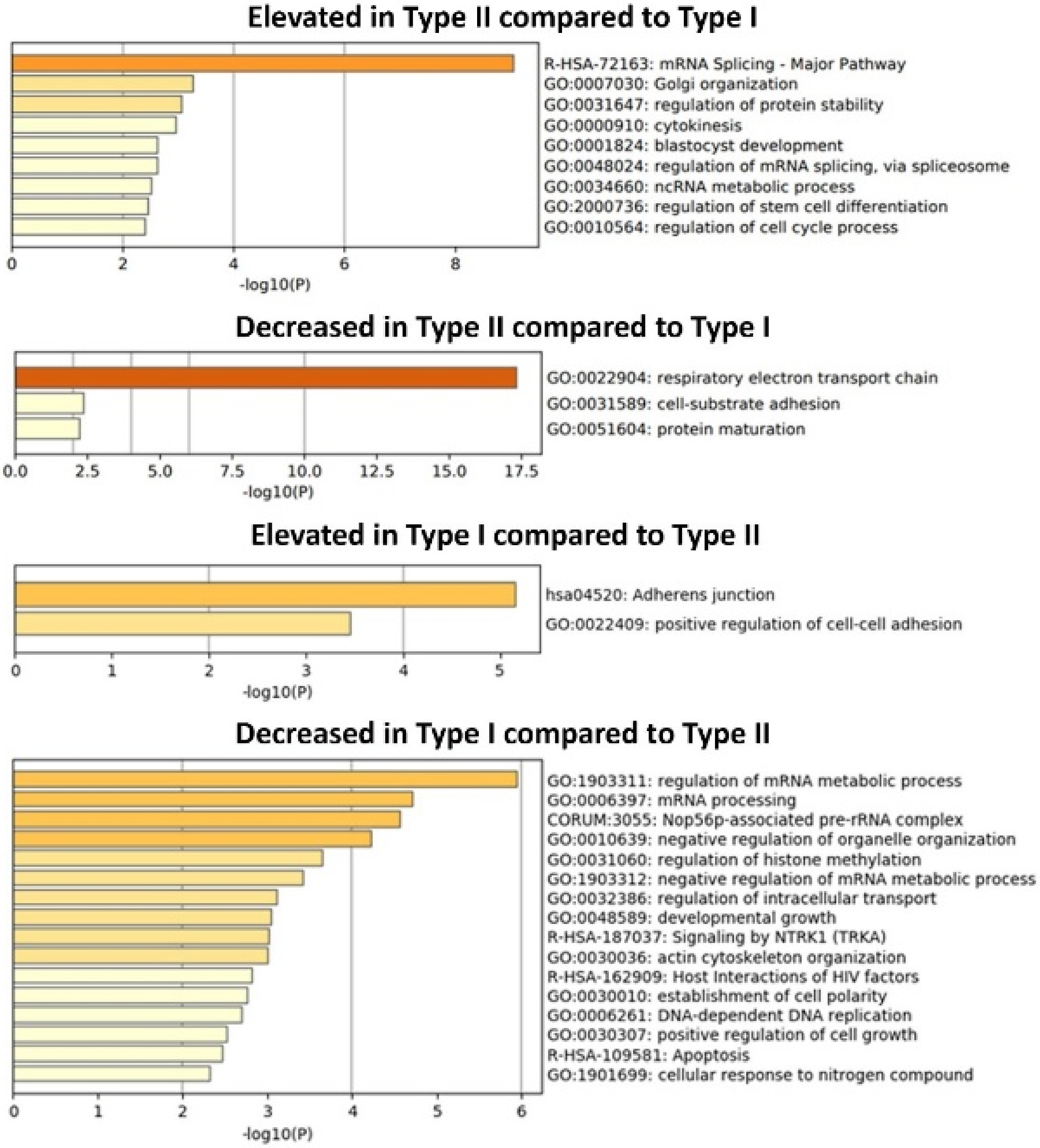

© 2022 Handley KF et al. *JAMA Network Open.*

eFigure 3. Differential mRNA Transcript Expression Analysis of HGSOC Type I and Type II Tumors. Representative Gene Set Enrichment Analysis (GSEA) of RNA Sequencing Results Showing Pathway Changes in Primary (A) and Metastatic (B) Site Samples

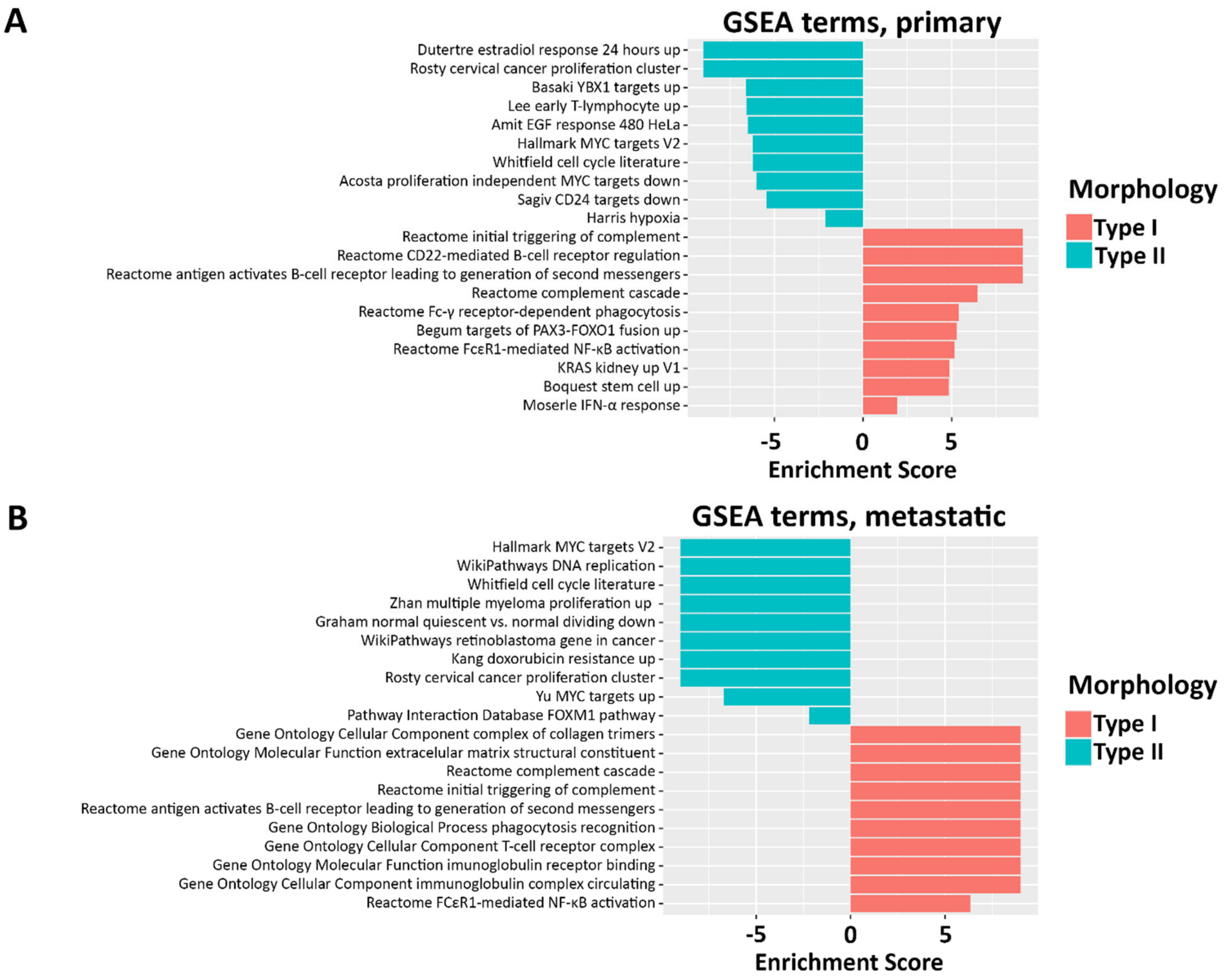

Enrichment scores are calculated based on GSEA reported p-values and false discovery rate values. Abbreviations: GSEA, Gene Set Enrichment Analysis; YBX1, Y-box binding protein 1; EGF, epidermal growth factor; MYC, avian myelocytomatosis viral proto-oncogene; CD, cluster of differentiation; Fc-γ, fragment crystallizable-gamma; PAX3, paired box 3; FOXO1, forkhead box O1; FcεR1, fragment crystallizable-epsilon receptor 1; NF-κB, nuclear factor-kappa B; KRAS, Kirsten rat sarcoma viral proto-oncogene; IFN-α, interferon-alpha; FOXM1, forkhead box M1.

© 2022 Handley KF et al. *JAMA Network Open.*

eFigure 4. Volcano Plots from RNA Sequencing of Patient-Derived (A) Primary and (B) Metastatic High-Grade Serous Ovarian Cancer Samples of Type I vs Type II Morphologic Subtypes

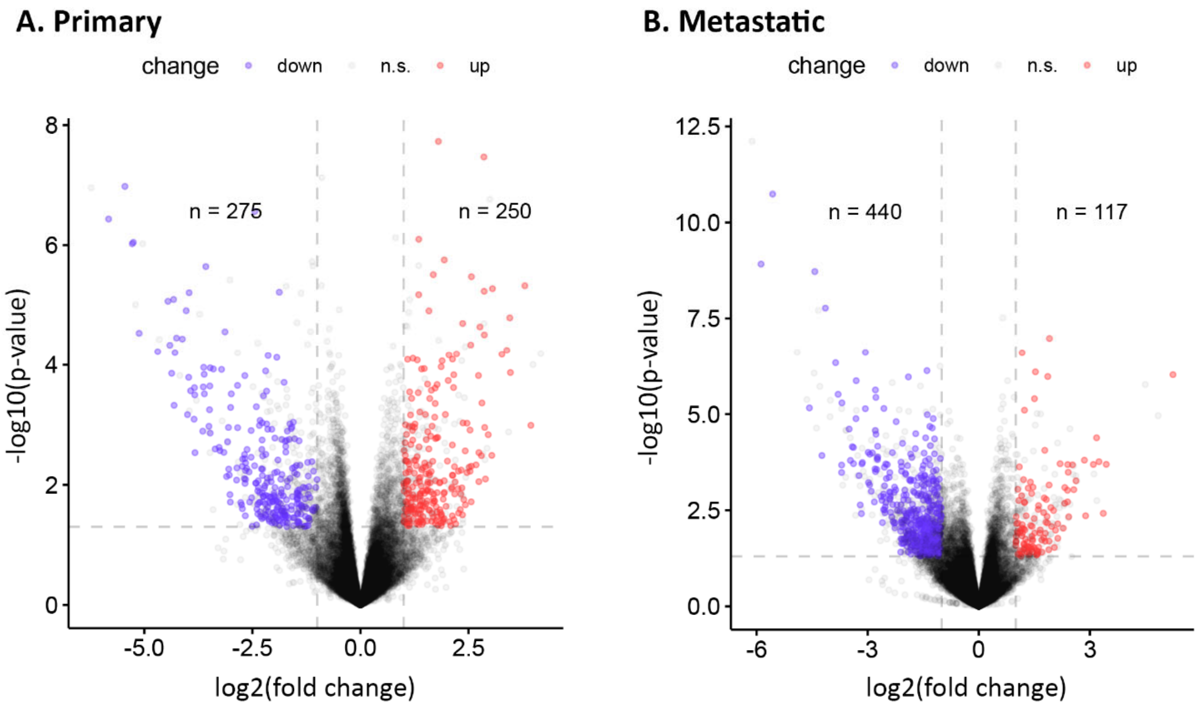

Thresholds for statistical significance, represented by the dashed lines, were $p < 0.05$ and $\log_2$ fold change of >1.0 or <-1.0.
Abbreviations: n.s., not significant

© 2022 Handley KF et al. *JAMA Network Open.*

eFigure 5. (A) Concordance Between All Genes and Corresponding Proteins Profiled by Both RNA Sequencing-Based Transcriptomics and Quantitative Mass Spectroscopy-Based Proteomics. (B) Correlation of Changes in Differentially Expressed Transcript/Protein Levels Between Type I and Type II Morphologic Subtypes

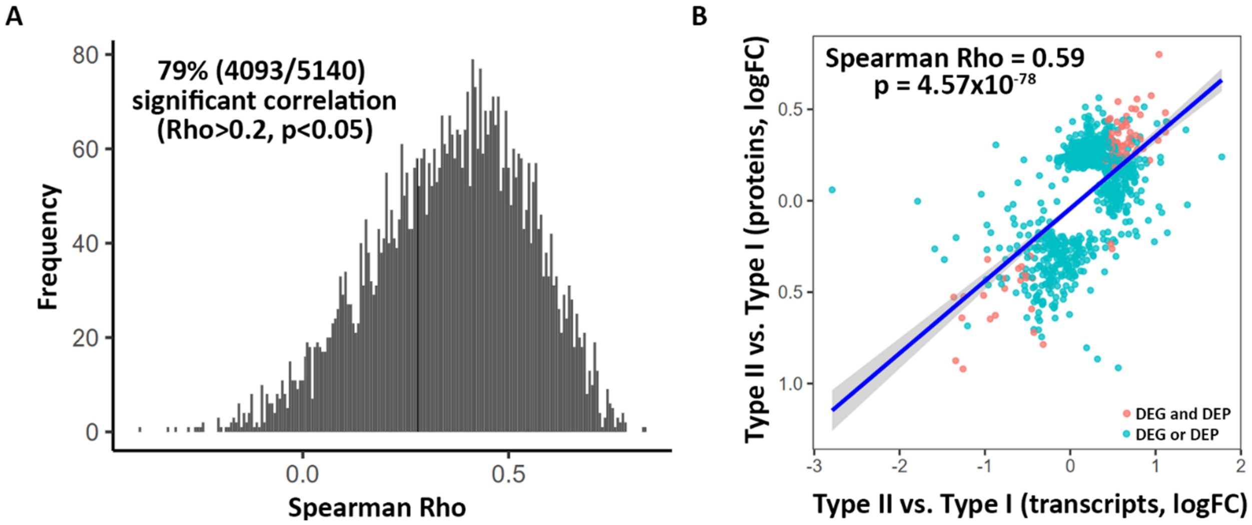

Red, genes identified by both RNA sequencing and quantitative mass spectroscopy. Blue, genes identified by either RNA sequencing or quantitative mass spectroscopy.
Abbreviations: FC, fold change; DEG, differentially expressed gene; DEP, differentially expressed protein

© 2022 Handley KF et al. *JAMA Network Open.*

eFigure 6. Immune Population Infiltration in Predominant Type I and Type II Morphologic Subtypes in the (A) Tumor Area and (B) Total Area (Tumor/Non-Tumor)

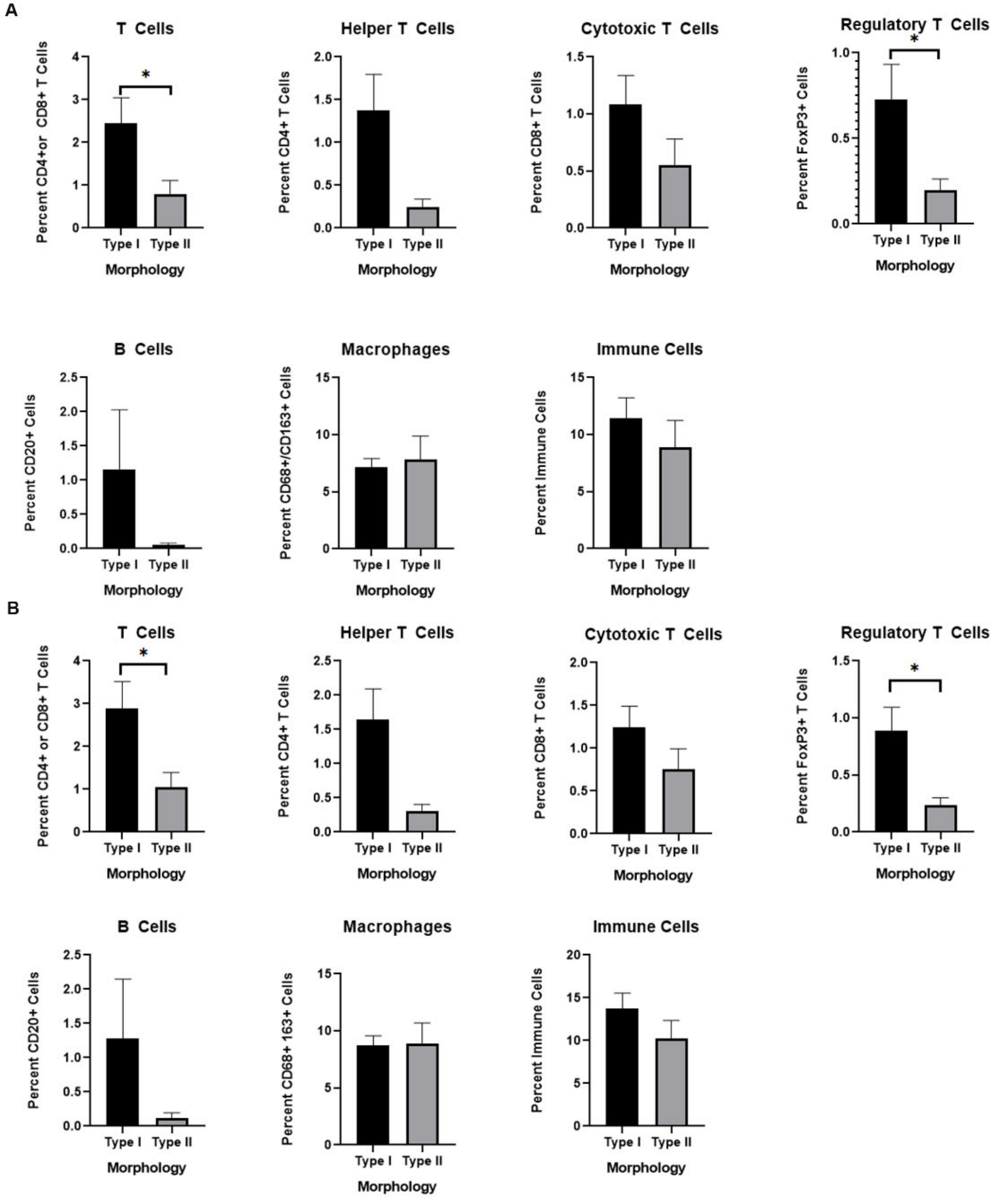

The percentages of immune populations, including all T cells, helper T cells, cytotoxic T cells, regulatory T cells, B cells, macrophages, and all immune cells, were compared. P-values were determined with either a Welch t-test or a Mann-Whitney test according to whether the data were normally distributed. Data are presented as the means ± SEMs. *p<0.05.

© 2022 Handley KF et al. *JAMA Network Open.*

eFigure 7. Representative Desorption Electrospray Ionization Mass Specta and Ion Images for Metastatic Tissues of Type I and Type II Morphologic Subtypes

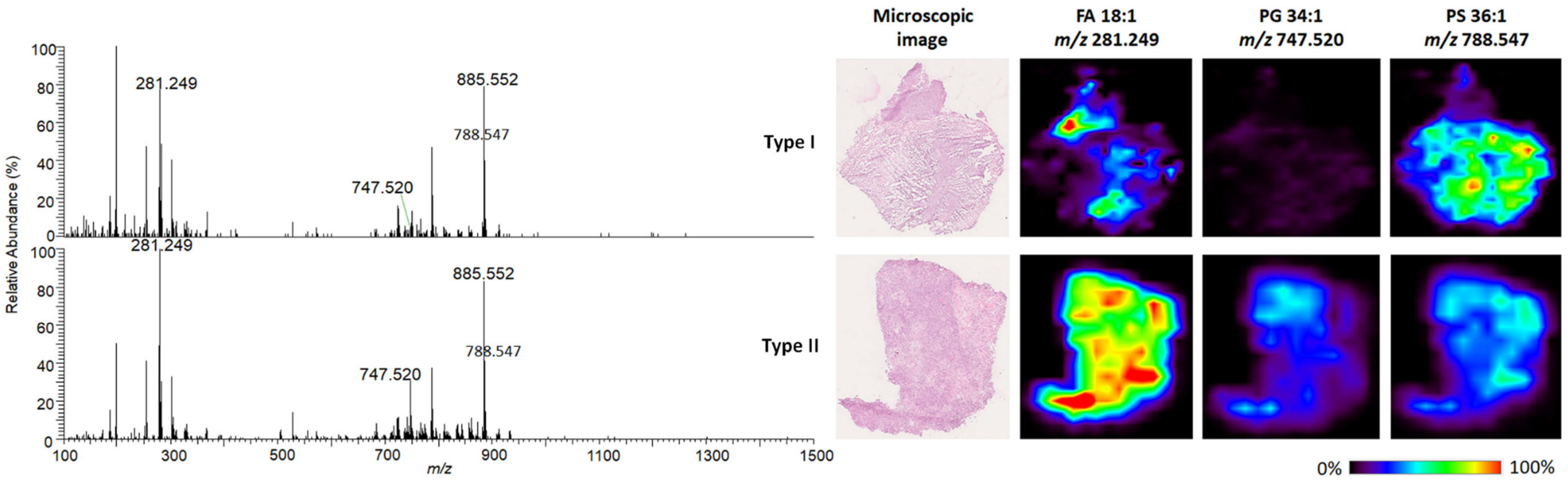

In the ion images, red areas represent the highest relative abundances (100%), and black areas represent the lowest relative abundances (0%). Lipid species are described by their numbers of fatty-acid chain carbons and double bonds.

© 2022 Handley KF et al. *JAMA Network Open.*

eReferences

1. Fleming ND, Nick AM, Coleman RL, et al. Laparoscopic Surgical Algorithm to Triage the Timing of Tumor Reductive Surgery in Advanced Ovarian Cancer. *Obstet Gynecol.* 2018;132(3):545-554.
2. Harris PA, Taylor R, Thielke R, Payne J, Gonzalez N, Conde JG. Research electronic data capture (REDCap)--a metadata-driven methodology and workflow process for providing translational research informatics support. *J Biomed Inform.* 2009;42(2):377-381.
3. Lee S, Zhao L, Rojas C, et al. Molecular Analysis of Clinically Defined Subsets of High-Grade Serous Ovarian Cancer. *Cell Rep.* 2020;31(2):107502.
4. Patro R, Duggal G, Love MI, Irizarry RA, Kingsford C. Salmon provides fast and bias-aware quantification of transcript expression. *Nat Methods.* 2017;14(4):417-419.
5. Zhang Y, Parmigiani G, Johnson WE. ComBat-seq: batch effect adjustment for RNA-seq count data. *NAR Genom Bioinform.* 2020;2(3):lqaa078.
6. Yoshihara K, Shahmoradgoli M, Martinez E, et al. Inferring tumour purity and stromal and immune cell admixture from expression data. *Nat Commun.* 2013;4:2612.
7. Subramanian A, Tamayo P, Mootha VK, et al. Gene set enrichment analysis: a knowledge-based approach for interpreting genome-wide expression profiles. *Proc Natl Acad Sci U S A.* 2005;102(43):15545-15550.
8. Mootha VK, Lindgren CM, Eriksson KF, et al. PGC-1alpha-responsive genes involved in oxidative phosphorylation are coordinately downregulated in human diabetes. *Nat Genet.* 2003;34(3):267-273.
9. Eberlin LS, Tibshirani RJ, Zhang J, et al. Molecular assessment of surgical-resection margins of gastric cancer by mass-spectrometric imaging. *Proc Natl Acad Sci U S A.* 2014;111(7):2436-2441.
10. Eberlin LS, Ferreira CR, Dill AL, Ifa DR, Cheng L, Cooks RG. Nondestructive, histologically compatible tissue imaging by desorption electrospray ionization mass spectrometry. *Chembiochem.* 2011;12(14):2129-2132.

© 2022 Handley KF et al. *JAMA Network Open.*
